# The Economic and societal burden associated with drug-resistant epilepsy in the Netherlands: an AIM@EPILEPSY burden-of-disease study protocol

**DOI:** 10.1136/bmjopen-2024-095123

**Published:** 2025-07-25

**Authors:** Darin Elabbasy, Silvia Evers, Marian H J M Majoie, Olaf E M G Schijns, Laura M’Rabet, Vivianne H J M van Kranen-Mastenbroek, Daniëlle B P Eekers, Ruud Houben, Marc Hendriks, Albert Colon, Ghislaine A P G van Mastrigt

**Affiliations:** 1 Department of Health Services Research, Maastricht University Care and Public Health Research Institute, Maastricht, Limburg, The Netherlands; 2 Trimbos Institute, Utrecht, The Netherlands; 3 Department of Neurology, Mental Health and Neuroscience Research Institute, Maastricht, Limburg, The Netherlands; 4 Academic Center for Epileptology, Maastricht University Medical Center and Kempenhaeghe, Heeze and Maastricht, The Netherlands; 5 Department of Neurosurgery, Maastricht University Medical Centre+, Maastricht, Limburg, The Netherlands; 6 Mental Health and Neuroscience Research Institute, Maastricht University, Maastricht, Limburg, The Netherlands; 7 MT Member Knowledge and Innovation, EpilepsieNL, Utrecht, The Netherlands; 8 Department of Clinical Neurophysiology, Maastricht University Medical Centre+, Maastricht, The Netherlands; 9 Department of Radiation Oncology (Maastro), GROW School for Oncology and Reproduction, Maastricht University Medical Centre+, Maastricht, Limburg, The Netherlands; 10 Donders Institute for Brain, Cognition and Behaviour, Radboud Universiteit, Nijmegen, Gelderland, The Netherlands; 11 Centre des Etudes et Traitements de l’ Epilepsie de la Caraïbe (CETEC), CHU-Martinique, Fort de France, France

**Keywords:** Epilepsy, Quality of Life, Caregiver Burden, Health Care Costs

## Abstract

**Abstract:**

**Background:**

Living with epilepsy, especially drug-resistant epilepsy (DRE), imposes several challenges for people diagnosed with the condition. These challenges include the physical and mental implications of epilepsy on both caregivers and patients with epilepsy. For the more than 120 000 individuals living with this neurological disorder in the Netherlands, along with their families, daily activities become hazardous, limited and costly, significantly affecting their health-related quality of life (HRQoL). As data on the burden of epilepsy in the Netherlands are lacking, studies attempting to capture the impact of epilepsy on individuals, caregivers and society are needed to enhance understanding and help address the burden of epileptic seizures.

**Methods and analysis:**

The study is part of the AIM@EPILEPSY project. The project aims to develop a planning suite enabling cost-saving, minimally invasive treatment for epilepsy. By surveying 330 people with epilepsy and an anticipated sample of 150–200 informal caregivers across the Netherlands, using standardised questionnaires focusing on associated societal costs and the impact on HRQoL, this bottom-up, prevalence-based prospective study aims to understand the societal burden of DRE in the Netherlands. The data will be collected at 0, 3, 6 and 12 months of follow-up. The study results will describe the economic impact of epilepsy, focusing on cost-of-illness (€) and HRQoL (utilities) in the Netherlands.

**Ethics and dissemination:**

The proposed study was approved by the Maastricht University Medical Ethics Review Committee (Approval reference: FHML-REC/2024/067/Amendment/2024_16). The result of the study is planned to be published in a peer-reviewed journal and presented at international and local scientific conferences.

**STRENGTHS AND LIMITATIONS OF THIS STUDY**

- The planned study will use a bottom-up costing approach from a societal perspective to quantify epilepsy-related costs.
- Data on quality of life and cost of illness will be collected using both disease-specific and generic instruments.
- The proposed study design may introduce selection and recall bias due to voluntary participation and self-reported data.

## Introduction

 This study is part of the ‘AIM@EPILEPSY: AI-assisted 4D cortical assessment for minimally invasive treatment of drug-resistant focal epilepsy’ project. The AIM@EPILEPSY project is developing new artificial intelligence-assisted methods for epileptogenic zones (EZ) delimitation with the goal of enhancing the accuracy of epilepsy surgeries and diagnosis.

Epilepsy is considered an umbrella term which encompasses a spectrum disorder with a wide range of severities and seizure types. The majority of people with epilepsy have a chronic form of epileptic seizures^1^ that are usually unpredictable and can pose dangerous effects on patient safety, increasing the risk of injury, hospitalisation and mortality.^2^ Beyond the physical consequences, it is also reported that people with epilepsy experience other comorbidities, including mental disorders such as depression, anxiety, psychosis and heightened susceptibility to suicidal ideations.^3^

These overlapping conditions not only sophisticate the medical management of epilepsy but also have a substantial impact on patients’ health-related quality of life (HRQoL).^4^ Consequently, epilepsy has significant implications for individuals’ health and well-being, affecting their ability to function in society. Moreover, the family and relatives of people with epilepsy experience a burden of caregiving, which includes emotional, psychological, physical and economic impacts, as well as distressing feelings such as shame, embarrassment, anger, guilt and, in some cases, self-blame.^5^

The first-line treatment for epilepsy is anti-seizure medication (ASM), but globally, one-third of people with epilepsy experience drug resistance (also known as medically refractory epilepsy or pharmaco-resistant epilepsy, hereafter referred to as DRE).^6^ DRE is defined as a condition in which an individual continues to experience seizures despite having undergone sufficient trials of two different ASMs.^7^

As quality of life is closely related to managing seizure frequency and occurrences,^4^ the negative impact of epilepsy on HRQoL is even more critical in people with DRE.^6^ People with DRE rely heavily on their family and friends for daily support, which can limit their independence and participation in everyday activities.

The other possible treatment option for people with DRE is to undergo epilepsy surgery. This surgical option involves the removal of the EZ, possibly helping people with epilepsy to achieve seizure freedom.^8^ However, 40%–50% of people with DRE, despite the meticulous and strict selection criteria for surgery, experience unfavourable outcomes.^9^ Moreover, since this surgery depends on the precise delineation of the EZs, there remains a pressing need for more advanced techniques to precisely delineate EZs to treat people with DRE more effectively.^10 11^

Another possible intervention for patients ineligible for curative (re)surgery is neuromodulation, a palliative intervention aimed usually to prevent or reduce ictal events.^12^ These include invasive methods such as vagus nerve stimulation, deep brain stimulation and responsive neurostimulation, as well as non-invasive techniques including transcutaneous vagus nerve stimulation, transcranial magnetic stimulation, transcranial direct current stimulation and trigeminal nerve stimulation.^12^

Both DRE and controlled epilepsy create a social and economic burden for the affected individuals and their caregivers. Among the 50 million people with epilepsy globally, approximately 12 000 people in the Netherlands live with an active form of epilepsy.^13^ Similar to many other countries, the evidence in the Netherlands on the burden of epileptic seizures is fragmented, showing variability in how data are collected and reported. Accurate knowledge of the cost of illness (COI) and the impact of the burden of disease (BoD) on HRQoL is essential for formulating and prioritising healthcare policies and interventions and for allocating healthcare resources efficiently within budget constraints.^14^ More importantly, COI studies can also indicate which diseases would benefit most from novel interventions aimed at reducing the BoD. This is especially relevant for chronic diseases such as epilepsy, which inflict a significant burden on healthcare expenditure and where new interventions are needed.

To understand and address the burden of epileptic seizures, there is a need for comprehensive studies that capture the entire range of their effects on individuals, caregivers and society. Using a prospective prevalence-based and bottom-up method, this study aims to understand the societal impact of epilepsy in the Netherlands, specifically in terms of the associated societal costs and impact on HRQoL. Additionally, the study will investigate the costs sustained by caregivers as well as the HRQoL consequences for caregivers of people with epilepsy, generally known as ‘spillover effects’. This potential finding of the study will highlight the need for assistive technologies and policies that can improve the quality of life and the social costs for people with epilepsy.

## Methods and analysis

### Study design

This proposed prospective bottom-up prevalence-based BoD study will adopt a societal perspective to assess the burden of DRE expressed in COI (€) and HRQoL (utilities). The study will adhere to the guidelines for the critical appraisal of COI studies by Larg and Moss, the Dutch guidelines for costing studies and the methodological quality checklist for COI studies developed by Schnitzler and colleagues (online supplemental appendix A, quality checklist).^15^,^17^

In bottom-up (person-based) costing, costs are allocated based on actual resource use, including indirect costs, unlike top-down costing, in which researchers allocate costs to individual healthcare services based on overall healthcare spending and utilisation rates.^18^ This methodological difference in assessing costs can lead to less accurate cost estimates, making bottom-up costing a more detailed and accurate method for estimating healthcare costs from a societal perspective where all costs are considered regardless of who incurred them.^18^,^20^

BoD studies can adopt either a prevalence-based or an incidence-based approach to data collection. Prevalence-based evaluations assess the economic and societal impact of a disease over a specified period, usually a year.^14^ This approach is thus considered more efficient compared with the incidence-based approach.^21^ Furthermore, prevalence-based studies play an important role in highlighting the economic BoDs with limited or underestimated impact, such as epilepsy and DRE.^14^

### AIM@EPILEPSY

**Figure 1:**
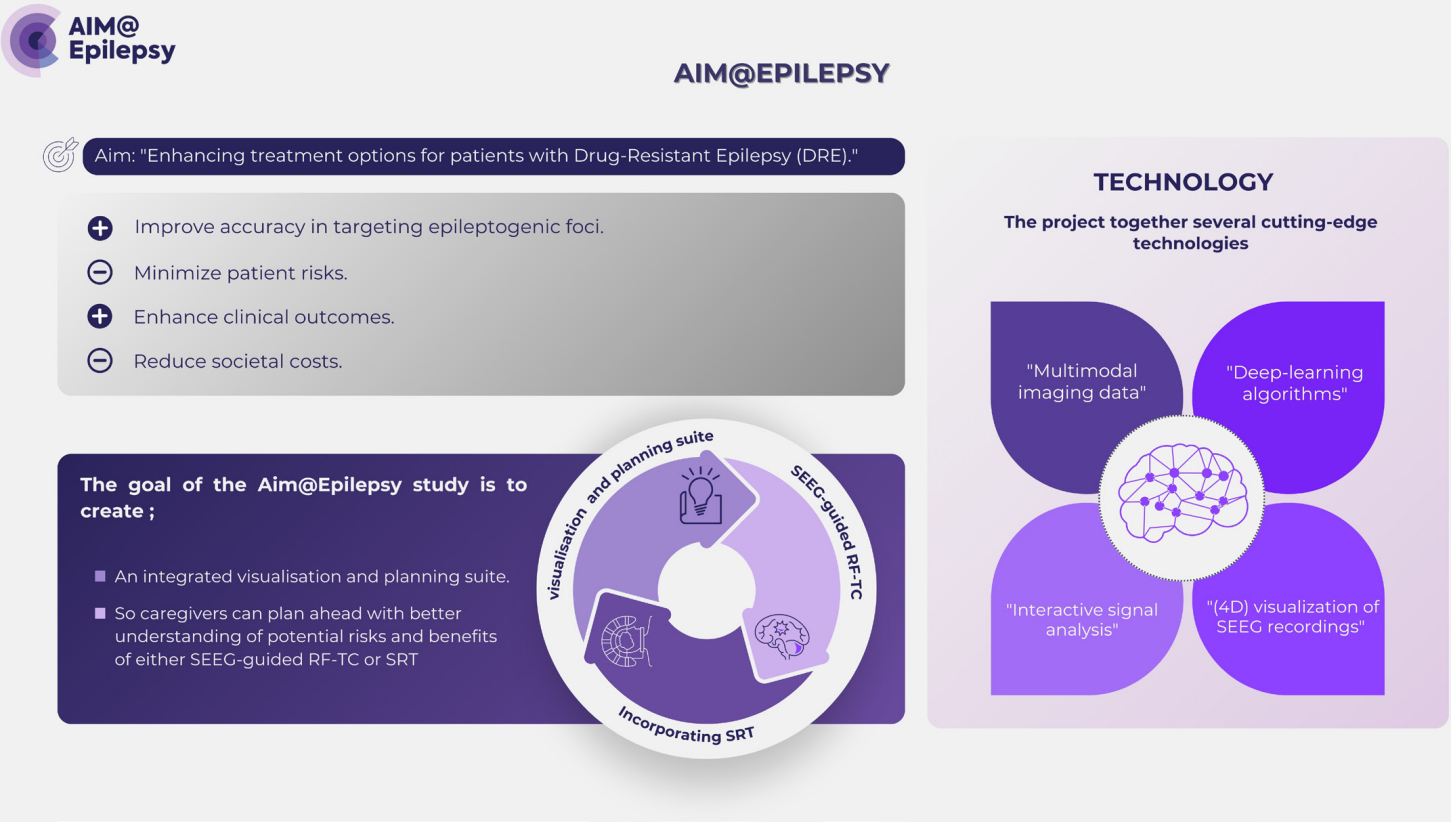
The AI-assisted 4D cortical assessment for minimally invasive treatment of epilepsy (AIM@EPILEPSY) project. 4D, four-dimensional; AI, artificial intelligence. SEEG-guided RF-TC, Stereo-Electro-Encephalography-guided RadioFrequency-ThermoCoagulation; SRT, Stereotactic Radiotherapy.

This study is a part of the AIM@EPILEPSY project. The project strives to improve treatment options for people with DRE, especially those who are not eligible for (re)surgery. AIM@EPILEPSY seeks to enhance the accuracy of targeting epileptogenic foci for minimally invasive curative treatment and therefore reduces patient risks, improves clinical outcomes and lowers societal costs (figure 1). The goal of the AIM@EPILEPSY project is to create an integrated visualisation and planning suite to support precision treatment using Stereo-Electro-Encephalography-guided RadioFrequency-ThermoCoagulation (SEEG-guided RF-TC) or Stereotactic Radiotherapy (SRT), which are key curative options for people with DRE. It integrates multimodal imaging data with deep-learning algorithms and interactive signal analysis with four-dimensional visualisation of invasive SEEG recordings. This combination helps medical professionals in treatment planning, enhancing recognition of potential risks and benefits of SEEG-guided RF-TC or SRT. This suite will potentially expand the pool of suitable people with DRE, minimise the complications associated with treatment, enhance the rates of successful outcomes and lessen both societal expenses and the overall impact of DRE.

### Participants and setting

To guarantee a representative sample, this study will be conducted in diverse settings. The study will take place at Maastricht University (MU), MU Medical Centre (MUMC+) in close collaboration with EpilepsieNL (the patient organisation for epilepsy in the Netherlands), The Academic Centre of Epileptology (Kempenhaeghe), and Maastro clinic.

This study will invite participants with the following criteria:

1. People with epilepsy:
  - Aged 18 and above, primarily residing in the Netherlands, fluent in Dutch and able to consent to participate.
  - A confirmed diagnosis of epilepsy by a neurologist (self-reported by study participants).
2. Individuals who are experiencing any type and frequency of epileptic seizures, which are defined according to the International League Against Epilepsy^7^ as ‘a transient occurrence of signs and/or symptoms due to abnormal excessive or synchronous neuronal activity in the brain’.
3. Adult caregivers of people with epilepsy: with caregivers, we refer to informal carers who provide unpaid assistance, such as help with personal hygiene, meal preparation or mobility, to a person with epilepsy within their immediate social circle. Caregivers of people with epilepsy or DRE who meet the inclusion criteria are eligible to complete a separate questionnaire assessing the caregiving burden.

Consequently, this study will exclude individuals under 18, individuals residing outside the Netherlands, or those with psychogenic non-epileptic seizures, which involve attacks that resemble epilepsy-related seizures but are not epileptic seizures, and individuals without the capacity to provide consent for the survey. For caregivers, we will exclude those who provide paid assistance to People with Epilepsy (PWE). No restrictions on diets or care received will be applied.

### Ethics and dissemination

The proposed study is approved by the Maastricht University Medical Ethics Review Committee (approval REF: FHML-REC/2024/067/Amendment/2024_16). The research team intends to publish both the study protocol and the study results in peer-reviewed scientific journals focused on health economics, health technology assessment and pharmacoeconomics, and present the planned study results in international and local scientific conferences.

Whether digitally through an electronic signature or in paper, all participants will receive an information letter and will be asked to sign an informed consent form before participating. The invitation to participate will make it clear that participants can withdraw from the study at any time without giving a reason. If a participant withdraws, their data will be deleted, unless it has already been fully de-identified and integrated into the analysis for publication.

### Public and patient involvement

The patient organisation EpilepsieNL was consulted during the planning and will be involved in the execution of this research. The data collection tool will be piloted by people with epilepsy before being disseminated to ensure its suitability and to ensure that the questions’ setup is appropriate for all participating individuals.

### Data collection and study sample

As no specific sample size calculation methods exist for BoD studies, and by referring to previously conducted studies with similar designs to ensure variation of the included population,^6 22^ we established our sample size of 330 people with epilepsy. In addition to recruiting people with epilepsy, we aim to recruit a separate sample of informal caregivers. While there is no fixed target sample size for caregivers, we expect to recruit approximately 150–200 caregivers to complete the survey.

For PWE and people with DRE, we will use a baseline survey along with two questionnaires on costs and three questionnaires on self-perceived HRQoL and well-being to aggregate data on the burden of DRE. Data will be prospectively collected using follow-up surveys at 0, 3, 6 and 12 months (table 1). For caregivers, two questionnaires will be administered: a baseline and another questionnaire specifically designed to measure and evaluate the impact of providing informal care.

**Table 1:** Overview of measurements timeline

	Time (months)			
	0	3	6	12
Baseline	x			
EQ-5D-5L (recall period: today)	x	x	x	x
iPCQ (adapted) (recall period: 4 weeks)	x	x	x	x
iMCQ (adapted) (recall period: 3 months)	x	x	x	x
QOLIE-31-P (recall period: 4 weeks)	x	x	x	x
ICECAP- A (recall period: now)	x	x	x	x
CarerQol-7D	x	x	x	x

See references.^25 26 36 42^

CarerQol-7D, The Care-related Quality of Life instrument; EQ-5D-5L, EuroQol questionnaire; ICECAP-A, ICEpop CAPability measure for Adults; iMCQ, iMTA Medical Consumption Questionnaire; iMTA, Institute for Medical Technology Assessment; iPCQ, iMTA Productivity Cost Questionnaire; QOLIE-31-P, The Patient Weighted Quality Of Life In Epilepsy.

The questionnaires will be available both digitally via the Qualtrics online survey tool (https://www.qualtrics.com/) and in paper formats between 29 October 2024 and 1 October 2026. The option to prevent multiple responses will be enabled for the digital questionnaire.

The partners (EpilepsieNL, Kempenhaeghe, MUMC+, Delft University of Technology (TU Delft), MU) and Maastro clinic’s social media platforms and websites will be used to invite the participants for the survey. Additionally, to ensure that we have reached many possible participants, we will create posters and flyers containing quick response (QR) codes with the link to the survey. Specialists, nurses and patient organisation staff from our partner organisations can hand out these materials and consent forms to people with epilepsy. The Qualtrics survey will also include digital informed consent. Once participants complete it, it will show a response summary, which they can download as a PDF. The patient and the researcher will have a copy of the informed consent for paper questionnaires (online supplemental appendix, informed consent and patient information sheet).

Before the survey is distributed, we will conduct a pilot study of face-to-face interviews in cooperation with EpilepsieNL to ensure that the questions’ length and setup are suitable for all participating patients. With their assistance and the use of their communication channels, we will invite individuals with epilepsy to participate in the survey pilot.

The participants will be asked to fill out the questionnaires, and after that, the researcher will ask the following questions:

- Did you feel like there were missing questions that should have been in the questionnaires?
- Was the questionnaire length suitable for you, or was it too long?
- And if it is too long, we will discuss with them their preferred survey length.
- Did you feel that the instructions for filling out the survey were clear?

The interviews will not be recorded (audio-taped). Nonetheless, the researcher will make sure that the surveys are completed by the piloting participants and will take note of the participants’ answers to the questions during the interviews. The information collected in the pilot study will not be included in the final analysis.

Furthermore, to ensure higher response and completion rates, all people with epilepsy who are willing to participate in the survey will be able to answer questions from the baseline questionnaire, which included the general questions regarding demographic data, the EuroQol 5-dimensions (EQ-5D-5L) quality of life questionnaire,^23^ The Institute for Medical Technology Assessment (iMTA) Medical Consumption Questionnaire (iMCQ)^24^ and iMTA Productivity Cost Questionnaire (iPCQ).^24^ Additionally, they will also be given the option to fill out the Patient Weighted Quality Of Life In Epilepsy (QOLIE-31-P)^25^ and the ICEpop CAPability measure for Adults (ICECAP-A).^26^ This will be achieved using Qualtrics’ display logic feature. After participants complete the EQ-5D-5L, iMCQ and iPCQ questionnaires, they will be prompted to decide whether they would like to proceed to answer additional questions (QOLIE-31-P and ICECAP-A). In the paper format, we will add a notice explaining the same procedure. Follow-up will be managed and monitored by the lead researchers in the study through Qualtrics.

### Baseline questionnaire

The baseline questionnaire will collect data on age, gender, living situation, contact information for follow-up, marital status, educational background, employment status, type of epilepsy (controlled with medication or DRE), age of first seizure and comorbid conditions. Questions about seizures will be sourced from the seizure diary questionnaire developed by Wester and colleagues (see online supplemental appendix B: PWE Questionnaires).^6^ These questions will cover the seizure frequency, the duration of each seizure episode and the emotional condition of the patient after a seizure occurs. Additionally, information regarding the year of initial diagnosis, current seizure status (active vs seizure-free) and level of independence in performing activities of daily living will be collected.

For caregivers, the baseline questionnaire will collect data on age, gender and employment status. In addition to questions related to epilepsy care recipients, and in line with other studies investigating the burden of care for epilepsy, we will collect information such as the caregiver’s relationship to the care recipient, the average hours spent per week providing care, and whether the caregiver has any underlying medical conditions.^6 27^

### Cost estimation

Bottom-up methods typically progress through three phases: identification, measurement and valuation of resources, in that sequence.^19^ To identify costs, we will document the healthcare resource usage of both people with epilepsy and their families at an individual level; cost categories will be employed to organise and delineate expenses and will be categorised into (1) healthcare costs, (2) patient and family costs and 3) cost to other sectors: productivity costs (table 2).^19^

**Table 2:** Cost categories for people with epilepsy

Healthcare sector costs	Patient and family costs	Cost to other sectors: productivity costs
General practitioner	Transportation	Presenteeism
Diagnostic tests	Specialised diets	Absenteeism
Primary care consultation	Informal care	
Medical interventions		
Rehabilitation		
Mental health services		
Assistive devices		
Medication		
Dietician		
Overnight stays		
Speech therapist		
Occupational physician/therapist		
Day treatment		
Homeopath/acupuncturist		
Emergency care		
Social worker		
Home care		
Outpatient care (<24 hours)		

To obtain data on healthcare utilisation and potential changes in productivity and patient and family costs, iMTA-iPCQ and iMTA-iMCQ will be used in this study. Both questionnaires are carefully developed and widely used in the Netherlands to evaluate healthcare consumption and productivity losses. They are also recommended in Dutch guidelines for the economic evaluation of health-related interventions.^17^ The iPCQ consists of three modules aimed at assessing productivity losses in paid work resulting from (1) absenteeism, (2) presenteeism, and it evaluates productivity losses associated with (3) unpaid work. The recall duration for the iPCQ is 4 weeks. Informal care and travel costs will also be captured using the adapted iMCQ questionnaire, where participants report the total hours of care received over 3 months from possibly multiple caregivers. This will be categorised into household activities, personal care and practical support. The value of these hours will be calculated using the replacement cost method.^28^ Travel costs related to healthcare providers’ visits will be reported, including mode of transportation, hospital visits and travel distance data. For other services, it will be assumed that participants travelled by car, using average distances from the Dutch costing manual for cost estimation.^28^

We will use the Dutch costing tool to assign monetary values to the healthcare services used, which provides reference prices for each resource.^28^ In instances where the Dutch costing manual did not provide reference prices, alternative sources will be used to derive the necessary pricing information. These will include the Dutch Healthcare Authority’s website (www.opendisdata.nl) for surgical procedures, the Dutch Healthcare Institute’s portal (www.gipdatabank.nl) for medical equipment and various supplier websites for non-medical equipment.^6^ Drug prices will be sourced from the Dutch Healthcare Institute’s website, available at www.farmacotherapeutischkompas.nl.

The costs associated with productivity loss due to presenteeism (working while unwell), and absenteeism (missing workdays) will be calculated. To assess the costs of presenteeism, we will take into account the number of work hours impacted, along with an efficiency score (rated on a numerical scale from 0 to 10) assigned by people with epilepsy based on their perceived work capability during those hours and factoring in the production loss costs per hour. To assess the cost of absenteeism, we will employ the friction cost method, which assumes that a replacement worker has fully taken over the responsibilities of the absent patient after a period of 85 days.^17^ Friction costs will be applied to people with epilepsy who are below the retirement age (67 years old).^29^ The combined total of costs attributed to both absenteeism and presenteeism will represent the overall costs of productivity loss related to paid work in the future. In cases where it is necessary, costs will be adjusted for inflation using the general price index provided by the Central Bureau of Statistics of the Netherlands (https://opendata.cbs.nl/statline/#/CBS/nl/dataset/70936ned/table). The final costs will be presented in 2024€.

### Health-related quality of life

Two validated questionnaires assessing self-perceived HRQoL; the EQ-5D-5L^30^ and the QOLIE-31-P)^25^ will be used in this study.

The EQ-5D-5L is a generic HRQoL instrument, which consists of five dimensions: mobility, self-care, usual activities, pain/discomfort and anxiety/depression. Responses from the descriptive system vary on a scale from 1, indicating no problems, to 5, signifying severe problems.^30^ Moreover, it included the Visual Analogue Scale (EQ-VAS), which is the second part of the questionnaire.^30^ The VAS scores range from 0, denoting the worst imaginable health state, to 100, representing the best imaginable health state.

Introduced by the ‘EuroQol Group’ the EQ-5D-5L is currently the most commonly used instrument for measuring HRQoL and is a reliable and valid instrument for the general population to value health outcomes.^31^,^33^ We will use the Dutch version of the EQ-5D-5L, and the utilities corresponding to the measured health states will be derived from the Dutch tariffs, with potential values ranging from −0.446 to 1.^34^ With 0 denoting ‘death’ and 1 representing ‘complete health’, negative values are indicative of health states deemed by the public to be a poorer state than death. Both the EQ-5D-5L and the EQ-VAS have a recall period of 1 day.^30^

Although the EQ-5D-5L questionnaire has a high adequacy and validity for assessing the quality of life in the general population and is recommended in the Dutch guidelines for economic evaluations,^23^ generic questionnaires could miss health impacts specific to epilepsy.^35^ Thus, we will be using the QOLIE-31-P,^25^ an epilepsy-specific questionnaire. The QOLIE-31-P is a 38-self-reported-item validated and translated to Dutch questionnaire, which assesses seven domains of epilepsy-related quality of life: seizure worry, overall quality of life, emotional well-being, energy/fatigue, cognitive functioning, medication effects and social functioning.^25^ Furthermore, each domain contains questions aimed at assessing the extent of distress an individual experiences with epilepsy. Raw scores from each subsection are transformed to a scale ranging from 0 to 100, with higher values being indicative of better HRQoL. The recall timeframe for the QOLIE-31-P is set to a duration of 4 weeks.

### Carer HRQoL

CarerQoL-7D and the CarerQoL-VAS (visual analogue scale) will be used to measure carer HRQoL. The Carer-QoL comprises seven sections that assess different dimensions of experiencing burden.^36^ The sections are satisfaction, support, problems with daily activities, and financial, relational, mental and physical health problems. For each dimension assessed, respondents can indicate whether they experience ‘no’ problems, ‘some’ problems or a lot of problems.

### Well-being

To assess the impact of epilepsy on well-being, we will use the ICECAP-A,^26^ which is a validated questionnaire designed to measure five capabilities related to the well-being of people with epilepsy. It differs from many profile measures in that it focuses on broader aspects of well-being rather than solely on health.^37^ The questionnaire shows good test-retest reliability and construct validity in a large Dutch sample.^38^ The index scores of the questionnaire will be calculated using the Dutch tariff of the Dutch translation of the ICECAP-A^39^ and will be scaled to range from 0 to 1, where ‘1’ indicates full capability and ‘0’ indicates no capability. The ICECAP-A has a recall period of ‘Now’.

### Data analysis

Descriptive statistics for all variables of interest will be calculated where continuous data will be described as mean, medians, SD and CIs, while categorical data will be presented as count (% and CIs). Data exploration will be conducted to assess the normality of the data, the presence of outliers and missing values. The normal distribution of HRQoL outcomes will be tested using the Shapiro-Wilk test. When the data is normally distributed, we will compute a parametric test, specifically an independent t-test. Conversely, when the data are not normally distributed, we will opt for a non-parametric test, such as the Mann-Whitney U test or Kruskal-Wallis in the case of multiple groups. A χ^2^ test will be carried out for categorical variables (or Analysis of Variance “ANOVA” in the case of multiple groups).

We will take into account that normality assumptions will be violated for cost data, and thus, we will perform a non-parametric bootstrapping using Excel (2019) software at 1000 replications for all cost categories. The alpha level will be set at 0.05 for all cost analyses. Differences in utility measurements that hold clinical significance will be determined by calculating half of the SD from a baseline measurement: a minimally important difference (MID) of 0.5 SD.^40^ All statistical analyses will be performed using R (V.4.0.2, R Foundation for Statistical Computing, Vienna, Austria)

### Sensitivity and subgroup analyses

To assess methodological uncertainties, we will conduct two sensitivity analyses; the first is by employing the UK tariff instead of the Dutch tariff.^17^ Second, to further examine the impact of differing viewpoints on cost outcomes, we will also estimate costs from a healthcare perspective as opposed to a societal perspective. A subgroup analysis will be conducted on all outcomes, including gender, age, level of education, seizure frequency, type of epilepsy (in terms of response to seizure control treatment) and work status.

### Handling of missing data

It is anticipated that there will be missing data due to loss to follow-up, non-responses or invalid responses (online supplemental appendix I, missing data handling). Multiple imputation techniques will be used in our analysis to correct missing values and ensure that equal weight is given to all data subjects in the dataset. If we encounter a missing value in one of the domains for the ICECAP-A or the EQ-5D-5L,^26 33^ we will impute the missing scores using the mean score of the domain before and after that domain where data is missing. For missing data on the QOILE-31-P, the scoring manual will be followed.^41^ In the case of failure of a member of the sample to respond to the survey as a whole in follow-up, the index score will be imputed using the mean of those who completed the entire questionnaire. Missing data on medical consumption will be imputed based on the mean value reported by people with epilepsy in the same treatment group. Since the friction method will be applied, missing data in the long-term productivity will not be imputed to avoid double counting.

## Discussion

Our proposed study aims to investigate the burden of DRE from a societal perspective, including the care costs incurred by healthcare providers, people with epilepsy, patient caregivers and other sectors. Employing validated patient-reported questionnaires designed to assess the burden of illness in patients and caregivers, this study will have a 1 year follow-up to better capture changes in HRQoL, healthcare utilisation, informal care and productivity losses. Additionally, we are including caregivers of people with DRE to identify the magnitude of this burden and its impact on HRQoL. The results of this study have the potential to advocate for the urgent need to develop medications, assistive technologies and policies that can significantly enhance the quality of life for individuals living with epilepsy. Moreover, the aggregated values of costs and utilities of people with epilepsy and their caregivers will be used to inform and construct a model-based economic evaluation of the AIM@EPILEPSY project.

The proposed study will follow reporting guidelines for costing studies, which will ensure that the methodology is robust and that the results are reproducible.^15 17^ Additionally, this study will be a bottom-up study from a societal perspective, which will ensure that all costs are captured. Moreover, to ensure that the proposed study captures an accurate image of the quality of life of individuals living with epilepsy, both disease-specific and generalised validated questionnaires will be used.

Even though every effort will be made to ensure that this proposed study will be thorough, the results of the study will be interpreted in the context of certain limitations. A standardised patient-reported questionnaire will be used in this study, which can lead to recall bias. Another potential limitation is that BoD studies that examine a single condition tend to overestimate costs due to the attribution of comorbid conditions. To provide an accurate estimate of the burden of DRE, we used broad inclusion criteria to include a study population that is representative of the real-world situation. As participation will be voluntary, there is a risk of selection bias. Moreover, as this study included those who are aged 18 years and older, the younger age groups will be, therefore, under-represented.

## Supplementary material

10.1136/bmjopen-2024-095123 online supplemental file 1
