# Supplementary material for: The Economic and societal burden associated with drug-resistant epilepsy in the Netherlands: an AIM@EPILEPSY burden-of-disease study protocol
Source: BMJ Open. 2025 Jul 25;15(7):e095123. doi: 10.1136/bmjopen-2024-095123 (PMC12306250; doi:10.1136/bmjopen-2024-095123)
Supplement: online supplemental file 1 [file bmjopen-15-7-s001.pdf]

Appendix A

| Item | Question | Answer[a] | Supportive information |
|---|---|---|---|
| Study characteristics | | | |
| Question/objective | 1) Is a well-defined research question or objective stated? | | |
| Population | 2) Is the study population described? | | |
| Perspective | 3) a) Is (are) the chosen study perspective(s) stated? | | |
| | b) If so, is (are) the chosen study perspective(s) justified? | | |
| Methodology and cost analysis | | | |
| Epidemiological approach | 4) Is the epidemiological approach reported (e.g., prevalence, incidence)? | | |
| Costing approach | 5) Is the costing approach reported (e.g., top-down, bottom-up)? | | |
| Data collection approach | 6) Is the data collection process reported (e.g., prospective, retrospective)? | | |
| Identification | 7) a) Are all components of resource use identified that are relevant to the condition/disease, population, intervention, study objectives, and study perspective? | | |
| | b) If not, is a justification provided for excluding relevant components of resource use? | | |
| Measurement | 8) a) Are all included components of resource use measured? | | |
| | b) If not, is a justification provided for not measuring certain components of resource use? | | |
| Valuation | 9) a) Are all included components of resource use valued in monetary terms? | | |
| | b) If not, is a justification provided for not valuing certain components of resource use? | | |
| Time horizon | 10) a) Is the chosen time horizon specified? | | |
| | b) If so, is the chosen time horizon justified? | | |
| Discounting | 11) a) Are future costs discounted? | | |
| | b) If so, is a justification provided for the discount rate? | | |
| Sensitivity | 12) a) Are all variables whose values are uncertain subjected to sensitivity analysis? | | |
| | b) If so, is a justification provided for which variables are subjected to sensitivity analysis? | | |
| | c) Are analyses done on relevant subgroups? | | |
| Results and reporting | | | |
| Cost sectors | 13) Are the study results presented transparently by cost category/sector? | | |
| Generalizability | 14) Do the authors discuss the generalizability of study results (e.g., comparing the results to other patient/client groups or/in other settings)? | | |
| Limitations | 15) Do the authors discuss important limitations? | | |
| Ethical and distributional issues | 16) a) Do the authors discuss ethical issues? | | |
| | b) Do the authors discuss distributional issues? | | |
| Conflict of interest | 17) Do the authors report any potential conflicts of interest? | | |

**Proefpersoneninformatie voor deelname aan medisch-wetenschappelijk onderzoek (versie 1, 20-5-2024)**

Geachte heer/mevrouw,

Hartelijk dank voor uw interesse in deze studie. Uw antwoorden op de vragenlijst zullen ons helpen om beter inzicht te krijgen in de impact van epileptische aanvallen op mensen met epilepsie en hun mantelzorgers

Lees alstublieft de volgende informatie goed door:

**Achtergrond en doel van de studie**
Het doel van dit onderzoek is om de kwaliteit van leven en de economische impact van epileptische aanvallen op mensen met epilepsie en hun mantelzorgers te onderzoeken. De deelname aan dit onderzoek is vrijwillig en het al dan niet meedoen aan dit onderzoek heeft geen gevolgen voor u behandeling.

**Wie kan deelnemen aan deze studie?**
Bent u een volwassene van 18 jaar of ouder en gediagnosticeerd met epilepsie, of verzorgt u iemand in uw omgeving met epilepsie? Dan kunt u deelnemen aan deze studie.

**Kan ik me terugtrekken uit deze studie?**
Deelname aan deze studie is volledig vrijwillig en u kunt op elk moment besluiten om zich terug te trekken. Uw gegevens worden van de UM-servers verwijderd, zolang deze nog niet geanalyseerd of gepubliceerd zijn. Nadat de analyse is voltooid, zal het voor het onderzoeksteam niet mogelijk zijn om individuen binnen een groep te onderscheiden, omdat de gegevens samengevoegd, samengevat of opgenomen worden in (gepubliceerde) bevindingen. Het onderzoeksteam zorgt ervoor dat de gegevens niet herleidbaar zijn tot de deelnemers. Als u uw deelname wilt beëindigen, kunt u contact opnemen met de onderzoeker. De contact gegevens staan onderaan deze brief

**Wat houdt deelname in en hoe lang duurt deze studie?**

We sturen u gedurende een jaar elke drie maanden een vragenlijst op. In totaal vult u dus vier keer een vragenlijst in. Het beantwoorden van de vragenlijsten duurt ongeveer 20 tot 50 minuten per keer.

Als u epilepsie heeft, zullen we algemene vragen stellen, maar ook vragen over zorggebruik (zoals medicijngebruik, bezoek aan huisarts), werkproductiviteit, welzijn en de impact van epilepsie op uw kwaliteit van leven.

Als u een mantelzorger bent, zullen we u ook algemene vragen stellen en enkele vragen over de impact van het verlenen van de zorg op uw kwaliteit van leven (invultijd voor mantelzorger bedraagt 10 minuten per keer).

**Proefpersoneninformatie voor deelname aan medisch-wetenschappelijk onderzoek (versie 1, 20-5-2024)**

**Hoe nemen we contact met u op?**

We sturen vervolgvragenlijsten op naar het opgegeven contactgegevens. Als de eerste vragenlijst niet meteen wordt ingevuld, ontvangt u een herinnering na 2 weken, gevolgd door een tweede herinnering na nog eens 2 weken. Als na deze herinneringen geen reactie is, volgt geen verdere contactpoging.

**Wat zijn de voordelen van deelname aan de studie?**

Uw deelname helpt onderzoekers waardevolle gegevens te verzamelen om de impact van epilepsie op de maatschappij maar ook de persoonlijke impact ervan beter te begrijpen. Door bij te dragen aan dit onderzoek, kunnen we de kwaliteit van de zorg voor mensen met epilepsie verbeteren.

**Is een vergoeding voor deelname aan de studie?**

Nee, deelname aan de studie is vrijwillig en er wordt geen vergoeding gegeven.

**Vertrouwelijkheid en delen van gegevens**

We voldoen aan de AVG-wetgeving (Algemene Verordening Gegevensbescherming) om uw privacy te beschermen.-Alleen onderzoekers die bij de studie betrokken zijn, hebben toegang tot uw gegevens. Door deel te nemen aan de studie stemt u in met het verzamelen, gebruiken en opslaan van uw gegevens. We zullen alle gegevens opslaan voor 15 jaar. Deze gegevens zullen worden gebruikt om onderzoeksvragen in dit onderzoek te beantwoorden maar ook voor eventuele vervolgonderzoeken. De data die we publiceren zullen nooit te herleiden zijn naar individuele personen.

**Wat gebeurt als u de vragenlijst niet volledig invult?**

Als u niet alle vragenlijsten invult, zijn er twee mogelijkheden. Ten eerste kan het zijn dat we de gegevens die u hebt verstrekt niet kunnen gebruiken omdat ze onvoldoende informatie bevatten. Ten tweede, de data die u heeft ingevuld bevat voldoende informatie en kan worden meegenomen in het onderzoek. We zullen voorafgaande aan de analyse pas de beslissing nemen over het al dan niet meenemen van onvolledige data. Het liefs willen we graag zoveel mogelijk complete data verzamelen en we hopen dan ook dat u alle vragenlijsten wilt invullen voor ons. Als u meer vragen hebt over dit onderwerp, neem dan contact op met de onderzoekers via de contactgegevens in deze brief.

**Als u vragen of verzoeken heeft, kunt u contact opnemen met:**


- Darin Elabbasy, Functie: Onderzoeker en PhD-kandidaat E-mailadres: darin.elabbasy@maastrichtuniversity.nl Telefoon: +31642914622,
- Dr. Ghislaine Van Mastrigt, Maastricht University, Care and Public Health Research Institute (CAPHRI).
- Prof Dr HJM Majoie MHM, neuroloog, Academisch Centrum voor Epileptologie, Kempenhaeghe & Maastricht UMC+

**Proefpersoneninformatie voor deelname aan medisch-wetenschappelijk onderzoek (versie 1, 20-5-2024)**

- Prof Silvia M.A.A. Evers, Maastricht University, Care and Public Health Research Institute (CAPHRI).

**Informatie over resultaten van onderzoek**

Indien gewenst kunnen we na afloop van dit onderzoek als we alle resultaten hebben u hierover informeren. U kunt aangegeven als u dit wilt.

**Toestemmingsformulier deelnemers.**

Na het lezen van bovenstaande informatie kunt u ervoor kiezen om deel te nemen aan deze studie.

Wilt u in de tabel hieronder ja of nee aankruisen

- Ik wil meedoen aan dit onderzoek. Ja ☐ Nee ☐
- Ik weet dat meedoen vrijwillig is. Ook weet ik dat ik op ieder moment kan beslissen om toch niet mee te doen met het onderzoek. Of om ermee te stoppen. Ik hoef dan niet te zeggen waarom ik wil stoppen. Ja ☐ Nee ☐
- Ik geef toestemming voor het bewaren van mijn gegevens voor een periode van 15 jaar. Ja ☐ Nee ☐
- Na afloop van de studie wil ik graag geïnformeerd worden over de resultaten van de studie. Ja ☐ Nee ☐
- Ik geef toestemming om mijn gegevens te bewaren om dit te gebruiken voor ander onderzoek, zoals in de informatiebrief staat. Ja ☐ Nee ☐

Appendix I

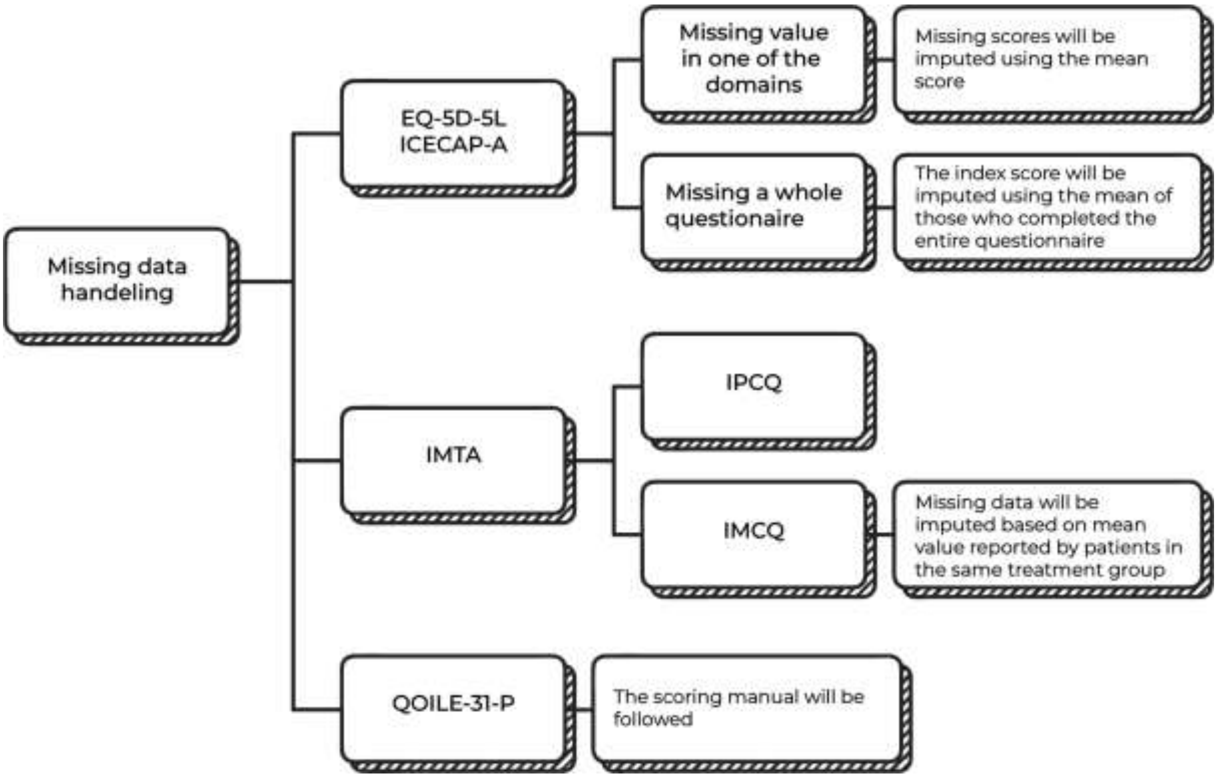
Missing data handeling
EQ-5D-5L ICECAP-A
Missing value in one of the domains
Missing scores will be imputed using the mean score
Missing a whole questionaire
The index score will be imputed using the mean of those who completed the entire questionnaire
IMTA
IPCQ
IMCQ
Missing data will be imputed based on mean value reported by patients in the same treatment group
QOILE-31-P
The scoring manual will be followed

Appendix B

# AIM@EPILEPSY - Main Survey

Page Break

*

Wat is uw naam? (Dit is optioneel)

________________________________________________________________

Page Break

*

Wat is uw e-mail?

________________________________________________________________

Page Break

*

Wat is uw leeftijd?

________________________________________________________________

Page Break

Wat is uw geslacht?

- ○ Man (1)
- ○ Vrouw (2)
- ○ Dat zou ik liever niet willen zeggen. (3)

Page Break

Wat is uw burgerlijke staat?

- ○ Alleenstaand (1)
- ○ Gehuwd / samenwonend (2)
- ○ Gescheiden (3)
- ○ Weduwe / weduwnaar (4)

Page Break

Wat is uw woonsituatie?

- ○ Zelfstandig, alleen (1)
- ○ Inwonend bij familie (bijvoorbeeld bij uw ouders) (2)
- ○ Zelfstandig, met kinderen (3)
- ○ Woonvoorziening met begeleiding op afstand (begeleid – zelfstandig) (4)
- ○ Zelfstandig, met partner (5)
- ○ Woonvoorziening met 24-uurs begeleiding (6)
- ○ Zelfstandig, met partner en kinderen (7)
- ○ Ik heb een andere woonsituatie, namelijk (8) ________________________________________________

Page Break

Wat is de hoogst genoten opleiding die u met een diploma heeft afgesloten?

- ◯ Geen (1)
- ◯ Basisschool (lager onderwijs, speciaal onderwijs) (2)
- ◯ Lager beroepsonderwijs (huishoudschool, LTS, LEAO, LHNO, ITO, LLO) (3)
- ◯ Middelbaar algemeen voortgezet onderwijs (VMBO, MAVO, IVO, MULO, ULO) (4)
- ◯ Middelbare beroepsopleiding (MTS, UTS, MEAO, MHNO, INAS) (5)
- ◯ Hoger algemeen en voorbereidend wetensch. onderwijs (HAVO, VWO, HBS, MMS) (6)
- ◯ Hoger beroepsonderwijs (HBO, HTS, HEAO, HHNO) (7)
- ◯ Universiteit (WO, doctoraalopleiding, TH) (8)
- ◯ Anders, namelijk; (9) ________________________________________________

Page Break

**Welke van de volgende omschrijvingen geeft uw belangrijkste dagelijkse werksituatie het beste weer?** Kruis aan wat u de meeste tijd doet.

- ○ Ik zit op school, ik studeer (1)
- ○ Ik werk in loondienst (2)
- ○ Ik ben zelfstandig ondernemer (3)
- ○ Ik ben huisvrouw of huisman (4)
- ○ Ik ben werkzoekend (5)
- ○ Ik ben (langdurig) arbeidsongeschikt (6)
- ○ Ik ben met pensioen of pre-pensioen (7)
- ○ Ik doe iets anders, namelijk; (8) ________________________________________________

Page Break

**Epilepsie**

*Het volgende deel van de vragenlijst gaat over uw epilepsie en uw behandeling voor epilepsie.*

Page Break

**Op welke leeftijd had u uw eerste epileptische aanval?** *Toen ik ........... jaar oud was.*

Page Break

*

In welk jaar werd bij u epilepsie vastgesteld door een arts?

__________________________________________________________________

Page Break

Welke vorm van epilepsie is er bij u vastgesteld?

○ Gegeneraliseerd (1)

○ partieel / focaal (2)

○ partieel / focaal en gegeneraliseerd (3)

○ Dat weet ik niet (4)

○ Anders, namelijk; (5) ________________________________________________

Page Break

**Welke aanvalstypen heeft u?** U kunt meerdere antwoorden aankruisen.

- ▢ Tonisch-clonisch (grote aanval, insult) (1)
- ▢ Tonisch (2)
- ▢ Clonisch (3)
- ▢ Atonisch (valaanval) (4)
- ▢ Myoclonisch (5)
- ▢ Absence (6)
- ▢ Eenvoudig partiëel (focale aanval met intacte gewaarwording) (7)
- ▢ Complex partiëel (focale aanval met verminderde gewaarwording) (8)
- ▢ Psychogene niet-epileptische aanval (PNEA) (9)
- ▢ Dat weet ik niet (10)
- ▢ Anders, namelijk: (11) ________________________________________________

Page Break

**Bij wie bent u onder behandeling voor uw epilepsie?** Kruis hier uw hoofdbehandelaar aan. Dit is de zorgverlener die over uw behandeling gaat, zoals uw medicijnen.

- ○ Neuroloog of kinderneuroloog in een epilepsiecentrum (SEIN, Kempenhaeghe) (1)
- ○ Neuroloog of kinderneuroloog in een ander ziekenhuis (2)
- ○ Huisarts (3)
- ○ Kinderarts (4)
- ○ Ik ben niet onder behandeling voor epilepsie (5)
- ○ Ik ben bij iemand anders onder behandeling, namelijk: (6) ________________________________________________

Page Break

**Hoeveel verschillende anti-epilepsie medicijnen heeft u geprobeerd?** Voorbeelden van anti-epilepsiemedicijnen zijn Keppra, Depakine, Vimpat, Frisium, Tregretol,  Diphantoine, Trileptal en Lamictal.

◯ 1  (1)

◯ 2  (3)

◯ 3 of 4  (5)

◯ 5 of 6  (2)

◯ Meer dan 6  (4)

◯ Dat weet ik niet  (6)

Page Break

**Hoeveel verschillende anti-epilepsie medicijnen gebruikt u op dit moment?**

- ○ 1 (3)
- ○ 2 (5)
- ○ 3 (2)
- ○ Meer dan 3 (4)
- ○ Geen (1)
- ○ Dat weet ik niet (6)

Page Break

**Heeft u epilepsie-chirurgie gehad?**

○ Nee, ik kom niet in aanmerking voor epilepsiechirurgie (1)

○ Nee, ik zit in het vooronderzoek voor epilepsiechirurgie (2)

○ Nee, ik weet niet of ik in aanmerking kom voor epilepsiechirurgie (3)

○ Nee, ik heb geen belangstelling voor epilepsiechirurgie (4)

○ Ja, in het jaar .. (5) ________________________________________________

Page Break

Heeft u een nervus-vagus stimulator (NVS)? De nervus vagus is een van de hersenzenuwen. Bij nervus vagus stimulatie (NVS) leggen we een elektrode om de linker nervus vagus. De elektrode is een rond, metalen plaatje van zo'n vier centimeter doorsnede met een dikte van ongeveer vijf millimeter.

○ Nee (1)

○ Ja, sinds het jaar .. (2) ________________________________________________

Page Break

**Heeft u een deep brain stimulator (DBS)?** De deep brain stimulator wordt ook wel  diepe hersen stimulator genoemd.

○ Nee  (1)

○ Ja, sinds het jaar ..  (2) ________________________________________________

Page Break

**Volgt u momenteel een ketogeen dieet?** Het ketogeen dieet is een vetrijk en koolhydraatarm dieet dat voorgeschreven kan worden als behandeling voor epilepsie.

- ○ Nee (1)
- ○ Ja, sinds het jaar .. (2) ________________________________________________

Page Break

**Welke hulpmiddelen gebruikt u?** U kunt meerdere antwoorden aankruisen.

- ☐ Aanvalsdagboek of aanvalskalender (1)
- ☐ Arm-, pols- of hoofdband met aanvalsdetectie (bijvoorbeeld Embrace of Nightwatch) (2)
- ☐ Matras-sensoren (bijvoorbeeld Emfit, Epi-Care, Epi-watcher of Epi-Wet) (3)
- ☐ Saturatiemeter (bijvoorbeeld Nonijn 900) (4)
- ☐ Dwaaldetectoren (bijvoorbeeld mat-alarm, infrarood sensor of magnetisch deurcontact) (5)
- ☐ Alarmeringssysteem (bijvoorbeeld een alarmknop, trekkoord, of alarmketting) (6)
- ☐ Valbescherming (bijvoorbeeld een valhelm of bescherming rondom radiatoren) (7)
- ☐ Ik gebruik geen hulpmiddelen (8)
- ☐ Ik gebruik een ander hulpmiddel, namelijk: (9) ________________________________________________

Page Break

**Heeft u naast epilepsie, nog andere ziektes of aandoeningen?** U kunt meerdere antwoorden aankruisen.

- ▢ Ja, een andere neurologische aandoening (bijvoorbeeld migraine, Parkinson,MS) (1)
- ▢ Ja, een motorische beperking (bijvoorbeeld verlamming, loopstoornis) (2)
- ▢ Ja, een verstandelijke beperking of (vermoedelijke) ontwikkelingsachterstand (3)
- ▢ Ja, CVA of beroerte (4)
- ▢ Ja, hart- en vaatziekte (bijvoorbeeld hartfalen, hartritmestoornis) (5)
- ▢ Ja, kanker of nieuwvormingen (bijvoorbeeld hersentumor, longkanker, borstkanker) (6)
- ▢ Ja, luchtwegaandoening (bijvoorbeeld astma, COPD) (7)
- ▢ Ja, diabetes mellitus (suikerziekte) (8)
- ▢ Ja, reuma, chronische rugklachten of artrose (9)
- ▢ Ja, psychische stoornis (bijvoorbeeld angststoornis, depressie) (10)
- ▢ Ja, ontwikkelings-, leer- of gedragsstoornis (bijvoorbeeld autisme, dyslexie, ADHD) (11)
- ▢ Ja, andere ziekte of aandoening, namelijk, ... (12) ________________________________________________
- ▢ Nee, ik heb geen andere ziektes of aandoeningen naast epilepsie (13)

End of Block: Block 1 -AchtergrondAchtergrond

---

Start of Block: Block 2- EQ-5D-5L -Gezondheidsvragenlijst

**Gezondheidsvragenlijst    Zet bij iedere categorie in de lijst hieronder een kruisje in het hokje dat het best past bij uw gezondheid VANDAAG.**

Page Break

Kies wat het beste past bij uw gezondheid VANDAAG **MOBILITEIT**

- ○ Ik heb geen problemen met lopen (1)
- ○ Ik heb een beetje problemen met lopen (2)
- ○ Ik heb matige problemen met lopen (3)
- ○ Ik heb ernstige problemen met lopen (4)
- ○ Ik ben niet in staat om te lopen (5)

Page Break

Kies wat het beste past bij uw gezondheid VANDAAG
**ZELFZORG**

- ○ Ik heb geen problemen met mijzelf wassen of aankleden (1)
- ○ Ik heb een beetje problemen met mijzelf wassen of aankleden (2)
- ○ Ik heb matige problemen met mijzelf wassen of aankleden (3)
- ○ Ik heb ernstige problemen met mijzelf wassen of aankleden (4)
- ○ Ik ben niet in staat mijzelf te wassen of aan te kleden (5)

Page Break

Kies wat het beste past bij uw gezondheid VANDAAG **DAGELIJKSE ACTIVITEITEN** (bijv. werk, studie, huishouden, gezins- en vrijetijdsactiviteiten)

- ○ Ik heb geen problemen met mijn dagelijkse activiteiten (1)
- ○ Ik heb een beetje problemen met mijn dagelijkse activiteiten (2)
- ○ Ik heb matige problemen met mijn dagelijkse activiteiten (3)
- ○ Ik heb ernstige problemen met mijn dagelijkse activiteiten (4)
- ○ Ik ben niet in staat mijn dagelijkse activiteiten uit te voeren (5)

Page Break

Kies wat het beste past bij uw gezondheid VANDAAG
**PIJN/ONGEMAK**

- ○ Ik heb geen pijn of ongemak (1)
- ○ Ik heb een beetje pijn of ongemak (2)
- ○ Ik heb matige pijn of ongemak (3)
- ○ Ik heb ernstige pijn of ongemak (4)
- ○ Ik heb extreme pijn of ongemak (5)

Page Break

Kies wat het beste past bij uw gezondheid VANDAAG **ANGST/SOMBERHEID**

- ◯ Ik ben niet angstig of somber (1)
- ◯ Ik ben een beetje angstig of somber (2)
- ◯ Ik ben matig angstig of somber (3)
- ◯ Ik ben erg angstig of somber (4)
- ◯ Ik ben extreem angstig of somber (5)

Page Break

**We willen weten hoe goed of slecht uw gezondheid VANDAAG is. Kijk naar de schaalafbeelding van 0 tot 100 en schrijf in het vak hoe goed of slecht jouw gezondheid vandaag is.** 0 is het slechtst en 100 is het best.

Page Break

**Je hebt zojuist de vragen over je gezondheidstoestand ingevuld. Door je ervaringen te delen, draag je bij aan onderzoek dat beleid wil vormgeven, wat mogelijk invloed heeft op de middelen en ondersteuning die beschikbaar zijn voor mensen met epilepsie. Jouw perspectief kan het verschil maken bij het bevorderen van zinvolle veranderingen op zowel gemeenschaps- als beleidsniveau.**

End of Block: Block 2- EQ-5D-5L -Gezondheidsvragenlijst

---

Start of Block: Block 3 IPCQ- gezondheid en werk

**Vragenlijst over uw gezondheid en werk**

Page Break

De volgende vragen gaan over uw werk. Met andere woorden, over werk waarvoor u betaald krijgt. Heeft u geen betaalde baan? Kies dan nee om door te gaan naar de volgende vraag.

- ○ Ja, ik heb een betaalde baan (1)
- ○ Nee (2)

Page Break

Wat is uw beroep?

________________________________________________________________

Page Break

*

**Hoeveel uur per week werkt u?** Tel alleen de uren waarvoor u betaald wordt.

________________________________________________________________

Page Break

*

Hoeveel dagen in de week werkt u?

________________________________________________________________

Page Break

Bent u in de afgelopen 4 weken afwezig geweest van uw werk omdat u  ziek was?

○ Nee  (1)

○ Ja, ik ben ….. dagen afwezig geweest (Tel alleen de werkdagen in de afgelopen 4 weken)  (2) ________________________________________________

Page Break

**Was u langer dan de gehele periode van 4 weken afwezig van uw werk  doordat u ziek was?** Het gaat om een aaneengesloten periode van werkverzuim.

- ○ Nee  (1)
- ○ Ja  (2)

Page Break

Wanneer heeft u zich ziek gemeld? (Datum)

________________________________________________

Page Break

Waren er in de afgelopen 4 weken dagen waarop u wel gewerkt heeft,  maar tijdens uw werk last had van lichamelijke of psychische problemen?

- ○ Nee  (1)
- ○ Ja  (2)

Page Break

**Op hoeveel werkdagen had u tijdens uw werk last van uw lichamelijke  of psychische problemen?** Tel alleen de werkdagen in de afgelopen 4 weken.

________________________________________________________________

Page Break

**Op de dagen dat u last had, kon u misschien niet zoveel werk doen als normaal. Hoeveel werk kon u op deze dagen gemiddeld doen?** Kijk naar de cijfers hieronder. Een 10 betekent dat u op deze dagen net zoveel kon doen als normaal. Een 0 betekent dat u op deze dagen niets kon doen. Klik het goede cijfer aan.

○ 0 (0)

○ 1 (1)

○ 2 (2)

○ 3 (3)

○ 4 (4)

○ 5 (5)

○ 6 (6)

○ 7 (7)

○ 8 (8)

○ 9 (9)

○ 10 (10)

Page Break

**Toelichting** Ook bij **onbetaald werk**, kunt u last hebben van uw lichamelijke of psychische problemen. Soms kunt u daardoor minder doen. U kunt bijvoorbeeld niet goed voor de kinderen zorgen of vrijwilligerswerk doen. Of geen boodschappen doen of in de tuin werken. Daarover gaan de volgende vragen. **Waren er dagen waarop u minder onbetaald werk kon doen door uw lichamelijke of psychische problemen?** Het gaat om dagen in de afgelopen 4 weken.

○ Nee (1)

○ Ja (2)

Page Break

*

**Tijdens hoeveel dagen was dit zo?** Tel alleen de dagen in de afgelopen 4 weken.

________________________________

Page Break

*

Stel dat iemand, bijvoorbeeld uw partner, familielid of een bekende, u op deze dagen had geholpen. En al het onbetaalde werk wat u niet kon doen, voor u had gedaan. Hoeveel uur was die persoon hier op deze dagen dan gemiddeld mee bezig geweest?

________________________________________________________________

Page Break

**Dank u voor het delen van uw ervaringen met betrekking tot uw werk en gezondheid. Dergelijke informatie is belangrijk voor ons, vooral voor degenen van wie de werkcondities worden beïnvloed door aanvallen, om de impact van epilepsie op werk te schatten. Dit levert ons gegevens op die ons helpen te pleiten voor meer innovatieve behandelingen voor mensen met epilepsie**

**End of Block: Block 3 IPCQ- gezondheid en werk**

---

**Start of Block: Block 4- imcq Vragenlijst over uw zorggebruik**

**Vragenlijst over uw zorggebruik**
**Toelichting** Wij willen graag weten met welke dokters u in de afgelopen 3 maanden een afspraak had. Het gaat om afspraken voor uzelf. Ook andere zorgverleners tellen mee. Bijvoorbeeld de fysiotherapeut of neuroloog. **Waar gaat de vragenlijst over?** De vragenlijst gaat over uw zorggebruik in de afgelopen 3 maanden. We beginnen met algemene vragen. Bijvoorbeeld over uw geslacht en geboortedatum. Daarna stellen we vragen over uw zorggebruik. **Welke afspraken tellen mee?** Controles Afspraken omdat u een lichamelijke of psychische klacht had Afspraken waarbij de dokter bij u thuis kwam Telefonische afspraken Telefoontjes met de receptenlijn
**Wat telt niet mee?** Afspraken voor een ander, bijvoorbeeld voor uw kind Telefoontjes om een afspraak te maken
Weet u niet precies hoeveel afspraken het waren? Noteer dan op hoeveel het er ongeveer waren.

Page Break

Heeft u in de afgelopen 3 maanden afspraken gehad met huisarts of praktijkondersteuner?
Praktijkondersteuner wordt ook wel POH genoemd.

○ Nee (1)

○ Ja (2)

Page Break

Hoeveel afspraken had u in de afgelopen 3 maanden met uw huisarts en/of praktijkondersteuner (POH)?

○ ...... afspraken huisarts (1) ________________________________________________

○ ...... afspraken praktijkondersteuner (POH) (2) ________________________________________________

Page Break

Hoeveel afspraken had u in de afgelopen 3 maanden met een  maatschappelijk werker?

- ○ Geen enkele afspraak  (1)
- ○ …… afspraken  (2) ________________________________________________

Page Break

**Hoeveel afspraken had u in de afgelopen 3 maanden met een fysiotherapeut? Of met een caesartherapeut, therapeut mensendieck of een manueel therapeut?** Tel alle afspraken met deze therapeuten bij elkaar op.

- ◯ Geen enkele afspraak (1)
- ◯ …… afspraken (2) ________________________________________________

Page Break

Hoeveel afspraken had u in de afgelopen 3 maanden met een  ergotherapeut?

○ Geen enkele afspraak  (1)

○ …… afspraken  (2) ________________________________________________

Page Break

Hoeveel afspraken had u in de afgelopen 3 maanden met een  logopedist?

○ Geen enkele afspraak  (1)

○ …… afspraken  (2) ________________________________________________

Page Break

Hoeveel afspraken had u in de afgelopen 3 maanden met een diëtist?

- ◯ Geen enkele afspraak (1)
- ◯ …… afspraken (2) ________________________________________________

Page Break

Hoeveel afspraken had u in de afgelopen 3 maanden met de neuroloog?

________________________________

Page Break

Hoeveel afspraken had u in de afgelopen 3 maanden met een epileptoloog?

________________________________

Page Break

Hoeveel afspraken had u in de afgelopen 3 maanden met de neurochirurg?

________________________________

Page Break

**Hoeveel afspraken had u in de afgelopen 3 maanden met een homeopaat? Of met een acupuncturist?** Tel alle afspraken met deze zorgverleners bij elkaar op.

- ○ Geen enkele afspraak (1)
- ○ …… afspraken (2) ________________________________________________

Page Break

**Hoeveel afspraken had u in de afgelopen 3 maanden met een psycholoog? Of met een psychotherapeut of psychiater?** Tel alle afspraken met deze zorgverleners bij elkaar op.

○ Geen enkele afspraak (1)

○ …… afspraken (2) ________________________________________________

Page Break

Hoeveel afspraken had u in de afgelopen 3 maanden met de  bedrijfsarts?

○ Geen enkele afspraak  (1)

○ …… afspraken  (2) ________________________________________________

Page Break

Heeft u in de afgelopen 3 maanden hulp van de thuiszorg gehad?

○ Nee (1)

○ Ja (2)

Page Break

**Wat voor hulp van de thuiszorg heeft u gehad in de afgelopen 3  maanden?** U kunt meer dan 1 hokje aankruisen.

- ☐ (1) **Huishoudelijke hulp** voorbeeld: stofzuigen, bed opmaken, boodschappen doen
- ☐ **Verzorging van uzelf** voorbeeld: hulp bij douchen of aankleden  (2)
- ☐ (3) **Verpleging** voorbeeld: verband omdoen, medicijnen geven, bloeddruk meten

Page Break

Hoeveel weken heeft u deze thuiszorg gehad? Tel alle weken in de afgelopen 3 maanden bij elkaar op. Let op: *een periode van 3 maanden telt 13 weken.* Als u geen hulp heeft gehad, kunt u '0' invullen.

- ▢ **Huishoudelijke hulp:** … weken in de afgelopen 3 maanden (1) ________________
- ▢ **Verzorging van uzelf:** … weken in de afgelopen 3 maanden (2) ________________
- ▢ **Verpleging:** … weken in de afgelopen 3 maanden (3) ________________

Page Break

13. Hoeveel uur thuiszorg kreeg u in deze weken gemiddeld?

- ☐ **Huishoudelijke hulp:** gemiddeld … uur in de week (1)
- ☐ **Verzorging van uzelf:** gemiddeld … uur in de week (2)
- ☐ **Verpleging:** gemiddeld … uur in de week (3)

Page Break

Heeft u in de afgelopen 3 maanden medicijnen gebruikt?

- ○ Ja (1)
- ○ Nee (2)

**Welke medicijnen heeft u in de afgelopen 3 maanden gebruikt?** Met medicijnen bedoelen we alle medicijnen die u hebt gekregen op recept en geneesmiddelen die u hebt gekocht bij de apotheek of de drogist. U ziet eerst drie voorbeelden. Lijst alle medicaties voor zowel epilepsie als niet-epilepsie aandoeningen. **Let op:** pak de verpakking erbij! Daarop staat hoeveel u per keer deze medicijnen moest nemen. En hoe vaak u dat moest doen. **Heeft u meer of minder gebruikt? Vul dan in hoeveel u ook echt gebruikt heeft.**

| | Hoe heet het medicijn? (6) | Hoeveel heeft u per keer genomen? *Kijk op de verpakking* (2) | Hoe vaak op een dag heeft u dit gedaan? *Kijk op de verpakking* (3) | Op hoeveel dagen in de afgelopen 3 maanden heeft u het medicijn gebruikt? (4) |
|---|---|---|---|---|
| 1 (1) | | | | |
| 2 (2) | | | | |
| 3 (3) | | | | |
| 4 (4) | | | | |
| 5 (5) | | | | |
| 6 (6) | | | | |
| 7 (7) | | | | |

| | | | | |
|---|---|---|---|---|
| 8 (8) | | | | |
| 9 (9) | | | | |
| 10 (10) | | | | |

Page Break

**Hoe vaak bent u in de afgelopen 3 maanden op de spoedeisende  eerste hulp van een ziekenhuis geweest?** Een andere naam voor spoedeisende  eerste hulp is EHBO.

- ○ Geen enkele keer  (1)
- ○ … keer  (2) ________________________________________________

Page Break

**Hoe vaak bent u in de afgelopen 3 maanden met een ambulance naar het ziekenhuis gebracht?** Een andere naam voor ambulance is ziekenauto

- ○ Geen enkele keer (1)
- ○ … keer (2) ________________________________________________

Page Break

**Had u in de afgelopen 3 maanden een afspraak bij de polikliniek van  het ziekenhuis?** Het gaat om afspraken voor uzelf met een dokter. Bijvoorbeeld met de  cardioloog, reumatoloog of neuroloog.

- ○ Nee  (1)
- ○ Ja  (2)

Bij welke soorten dokters bent u in de afgelopen 3 maanden in het  ziekenhuis geweest? En hoe vaak?

| | Bij welke soort dokter bent u in het ziekenhuis geweest? (1) | Hoe vaak bent u in de afgelopen 3  maanden bij deze dokter geweest? (2) |
|---|---|---|
| 1 (1) | | |
| 2 (2) | | |
| 3 (3) | | |
| 4 (4) | | |
| 5 (5) | | |
| 6 (6) | | |
| 7 (7) | | |
| 8 (8) | | |
| 9 (9) | | |

Page Break

**Bent u in de afgelopen 3 maanden overdag in het ziekenhuis geweest voor een behandeling?** U bleef dus niet slapen. U kwam bijvoorbeeld voor een bloedtransfusie, nierdialyse of chemokuur.

- ○ Nee (1)
- ○ Ja (2)

Voor welke soort behandeling was dit? Was dit voor meer dan 1 soort  behandeling? Vul dan alle soorten behandelingen in.

| | Vul dan alle soorten behandelingen in. (1) |
|---|---|
| Behandeling 1 (1) | |
| Behandeling 2 (2) | |
| Behandeling 3 (3) | |
| Behandeling 4 (4) | |
| Behandeling 5 (5) | |
| Behandeling 6 (6) | |
| Behandeling 7 (7) | |
| Behandeling 8 (8) | |

Page Break

Hoeveel keer moest u in de afgelopen **3 maanden** voor deze  behandelingen naar het ziekenhuis?

| | Hoe vaak (1) |
|---|---|
| Behandeling 1 (1) | |
| Behandeling 2 (2) | |
| Behandeling 3 (3) | |
| Behandeling 4 (4) | |
| Behandeling 5 (5) | |
| Behandeling 6 (6) | |
| Behandeling 7 (7) | |
| Behandeling 8 (8) | |

Page Break

**Bent u in de afgelopen 3 maanden ergens anders geweest voor een behandeling overdag?** U bleef dus niet slapen. U ging bijvoorbeeld naar de dagopvang van een woon-/zorgcentrum of een psychiatrische instelling. Of naar de dagbehandeling van een revalidatiecentrum.

○ Nee (1)

○ Ja (2)

Page Break

**Wat voor instelling was dit?** Kruis het goede antwoord aan. U kunt meer dan 1 hokje aankruisen.

- ☐ Woon-/zorgcentrum (1)
- ☐ Revalidatiecentrum (2)
- ☐ Psychiatrische instelling (3)
- ☐ Een andere instelling, namelijk .... (4) ________________

Page Break

**Hoe vaak moest u hier in de afgelopen 3 maanden naartoe?** Heeft u bij  vraag 24 meer dan 1 hokje aangekruist? Vul dan hieronder voor iedere instelling in hoe vaak u er bent geweest

| | keer in de afgelopen 3 maanden (1) |
| --- | --- |
| Naar het woon-/zorgcentrum (1) | |
| Naar het revalidatiecentrum (2) | |
| Naar de psychiatrische instelling (3) | |
| Naar de andere instelling (4) | |

Page Break

**Heeft u in de afgelopen 3 maanden weleens in het ziekenhuis gelegen?** U moest dus blijven slapen. Bijvoorbeeld omdat u geopereerd was en niet meteen naar huis kon.

- ○ Nee (1)
- ○ Ja (2)

Page Break

Hoe vaak heeft u in de afgelopen 3 maanden in het ziekenhuis  gelegen?

○ …… keer in de afgelopen 3 maanden  (1)

Page Break

Hoe lang heeft u in het ziekenhuis gelegen? Heeft u meer dan 1 keer in het ziekenhuis gelegen in de afgelopen 3 maanden? Tel dan alle dagen bij elkaar op.

○ …… dagen in totaal in de afgelopen 3 maanden (1)

________________________________

Page Break

**Moest u in de afgelopen 3 maanden ergens anders blijven slapen  voor uw gezondheid?**
Bijvoorbeeld in een woon-/zorgcentrum, psychiatrische instelling of  revalidatiecentrum.

○ Nee  (1)

○ Ja  (2)

Page Break

**Wat voor instelling was dit?** U kunt meer dan 1 hokje aankruisen.

- ☐ Woon-/zorgcentrum (1)
- ☐ Revalidatiecentrum (2)
- ☐ Psychiatrische instelling (3)
- ☐ Een andere instelling, namelijk .... (4) ________________________________________________

Page Break

*

**Hoe lang bent u in deze instelling geweest?** Heeft u bij vraag 30 meer dan 1 hokje aangekruist? Vul dan hieronder voor iedere instelling in hoe lang u er bent geweest. **Bent u ergens meer dan 1 keer geweest in de afgelopen 3 maanden? Tel dan alle dagen bij elkaar op.**

| | **dagen in de afgelopen 3 maanden** (1) |
|---|---|
| Naar het woon-/zorgcentrum (1) | |
| Naar het revalidatiecentrum (2) | |
| Naar de psychiatrische instelling (3) | |
| Naar de andere instelling (4) | |

Page Break

**Heeft u in de afgelopen 3 maanden hulp gekregen van een familielid of een bekende vanwege uw lichamelijke of psychische problemen?**

- ○ Nee (1)
- ○ Ja (2)

Page Break

**Wat voor hulp van familieleden of bekenden heeft u gehad in de afgelopen 3 maanden?** U kunt meer dan 1 hokje aankruisen.

- ▢ **Huishoudelijke hulp** voorbeeld: stofzuigen, bed opmaken, boodschappen doen, klaarmaken van eten en drinken, verzorgen van kinderen (1)
- ▢ **Verzorging van uzelf** voorbeeld: hulp bij douchen of aankleden, hulp bij het eten en drinken of het geven van medicijnen (4)
- ▢ **Praktische hulp** voorbeeld: ondersteuning bij wandelen, het maken van uitstapjes of bezoekjes aan bekenden, bezoeken aan de huisarts of het ziekenhuis, het regelen van hulp of het regelen van financiële zaken (5)

Page Break

**Hoeveel weken heeft u deze hulp gehad**? Tel alle weken in de afgelopen 3 maanden bij elkaar op. *Let op: een periode van 3 maanden telt 13 weken*.

- ○ **Huishoudelijke hulp:** ... weken in de afgelopen 3 maanden (1) __________________________________________________________________
- ○ **Verzorging van uzelf:** ... weken in de afgelopen 3 maanden (2) __________________________________________________________________
- ○ **Praktische hulp:** ... weken in de afgelopen 3 maanden (3) __________________________________________________________________

Page Break

**Hoeveel uur hulp kreeg u in deze weken gemiddeld?**

- ○ **Huishoudelijke hulp:** gemiddeld ... uur in de week (1) ______________________________________________________________
- ○ **Verzorging van uzelf:** gemiddeld ... uur in de week (2) ______________________________________________________________
- ○ **Praktische hulp:** gemiddeld ... uur in de week (3) ______________________________________________________________

Page Break

**Welke wijze van vervoer heeft u gebruikt om van huis naar het ziekenhuis te gaan?**

- ○ Niet van toepassing (1)
- ○ Te voet (2)
- ○ Fiets (3)
- ○ Auto (4)
- ○ Openbaar vervoer (5)
- ○ Taxi (6)
- ○ Anders, namelijk (7) ________________________________________________

Page Break

**Wat was de enkele reisafstand tussen uw huis en het ziekenhuis?**

○ **Deze afstand bedroeg:** ...... kilometer (1)

Page Break

**Door deze vragenlijst in te vullen, helpt u ons inzicht te krijgen in de zorgondersteuning die u nodig heeft en gebruikt. Uw antwoorden dragen bij aan onderzoek dat gericht is op het verbeteren van zorgdiensten en behandelingen voor mensen met vergelijkbare gezondheidsproblemen. Elke informatie die u geeft, brengt ons dichter bij het pleiten voor betere zorg en middelen voor patiënten zoals u.**

Page Break

Het volgende gedeelte is optioneel. U kunt **"Volgende"** selecteren om door te gaan, of naar **"de laatste stap"** gaan.

○ Volgende (1)

○ De laatste stap (2)

End of Block: Block 4- imcq Vragenlijst over uw zorggebruik

---

Start of Block: Block 5-

**Heeft u vragen of opmerkingen?** Misschien heeft u nog vragen of opmerkingen? Schrijft u deze dan hieronder op.

________________________________________________________________

________________________________________________________________

________________________________________________________________

________________________________________________________________

________________________________________________________________

End of Block: Block 5-

---

Start of Block: Welkom bij het optionele deel van de vragenlijst

JS

End of Block: Welkom bij het optionele deel van de vragenlijst

---

Start of Block: Block 1-2 Optionele achtergrondvragen

Page Break

**Epilepsie : uw aanvalskenmerken**

Deze vragenlijst gaat over uw aanvallen. In deze vragenlijst, beschrijft u hoe uw aanvallen eruit zien. Als u meerdere soorten aanvallen (aanvalstypes) heeft vragen wij u om elk aanvalstype apart te beschrijven.

Page Break

Toelichting: Geef een beschrijving van uw aanvallen. Kunt u zelf geen goede beschrijving van uw aanvallen geven, dan kan iemand u eventueel helpen. Bijvoorbeeld een familielid. Heeft u meerdere aanvalsvormen, beschrijf dan de 3 meest voorkomende typen.

○ Aanvalstype A (1) ________________________________________________

○ Aanvalstype B (2) ________________________________________________

○ Aanvalstype C (3) ________________________________________________

**Aanvalstype A**

Heeft u bij deze aanval schokken over uw hele lichaam?

○ Ja (1)

○ Nee (2)

Page Break

Hoe vaak valt u als gevolg van dit aanvalstype?

- ○ Bijna altijd/ Altijd (1)
- ○ Vaak (2)
- ○ Soms (3)
- ○ Nooit (4)

Page Break

**Ontstaat er als gevolg van dit aanvalstype een van de volgende letsels?** Kruis één hokje aan: het meest ernstige.

- ○ Verbranding, diepe snijwonden, breuken (1)
- ○ Tongbeet of ernstige hoofdpijn (2)
- ○ Milde verwonding of milde hoofdpijn (3)
- ○ Geen verwonding (4)

Page Break

Hoe vaak is er bij dit aanvalstype sprake van urineverlies?

- ○ Bijna altijd/ Altijd (1)
- ○ Vaak (2)
- ○ Soms (3)
- ○ Nooit (4)

Page Break

Als dit aanvalstype gepaard gaat met een bewustzijnsdaling, voelt u dit lang genoeg van te voren aankomen om uzelf te beschermen?

- ○ Bijna altijd/ Altijd (1)
- ○ Soms (2)
- ○ Nooit (3)
- ○ Geen van deze opties: er is geen bewustzijnsverlies bij mijn aanvallen (4)
- ○ Geen van deze opties: mijn aanvallen komen alleen in slaap voor (5)

Page Break

Hoe lang duurt het voor u weer helemaal de oude bent?

- ◯ Minder dan 1 minuut (1)
- ◯ Tussen 1 en 10 minuten (2)
- ◯ Tussen 10 min en 1 uur (3)
- ◯ Tussen 1 en 3 uur (4)
- ◯ Meer dan 3 uur (5)

Page Break

**Komen bij dit type aanval de volgende verschijnselen voor?** Kruis één hokje  aan: het meest ernstige

- ○ Schreeuwen; dwalen en/of ontkleden  (1)
- ○ Lichte schokken/ bewegingen in delen van het lichaam  (2)
- ○ Geen  (3)

Page Break

**Indien u slechts één type aanval heeft, gaat u vriendelijk verder naar het volgende deel.**

○ Aanvalstype B (1)

○ Volgende Sectie (2)

Page Break

**Aanvalstype B**

Page Break

Heeft u bij deze aanval schokken over uw hele lichaam?

- ○ Ja (1)
- ○ Nee (2)

Page Break

Hoe vaak valt u als gevolg van dit aanvalstype?

○ Bijna altijd/ Altijd (1)

○ Vaak (2)

○ Soms (3)

○ Nooit (4)

Page Break

**Ontstaat er als gevolg van dit aanvalstype een van de volgende letsels?** Kruis één hokje aan: het meest ernstige.

- ○ Verbranding, diepe snijwonden, breuken (1)
- ○ Tongbeet of ernstige hoofdpijn (2)
- ○ Milde verwonding of milde hoofdpijn (3)
- ○ Geen verwonding (4)

Page Break

Hoe vaak is er bij dit aanvalstype sprake van urineverlies?

- Bijna altijd/ Altijd (1)
- Vaak (2)
- Soms (3)
- Nooit (4)

Page Break

Als dit aanvalstype gepaard gaat met een bewustzijnsdaling, voelt u dit lang genoeg van te voren aankomen om uzelf te beschermen?

- ○ Bijna altijd/ Altijd (1)
- ○ Soms (2)
- ○ Nooit (3)
- ○ Geen van deze opties: er is geen bewustzijnsverlies bij mijn aanvallen (4)
- ○ Geen van deze opties: mijn aanvallen komen alleen in slaap voor (5)

Page Break

Hoe lang duurt het voor u weer helemaal de oude bent?

- ◯ Minder dan 1 minuut (1)
- ◯ Tussen 1 en 10 minuten (2)
- ◯ Tussen 10 min en 1 uur (3)
- ◯ Tussen 1 en 3 uur (4)
- ◯ Meer dan 3 uur (5)

Page Break

**Komen bij dit type aanval de volgende verschijnselen voor?** Kruis één hokje aan: het meest ernstige

- ○ Schreeuwen; dwalen en/of ontkleden (1)
- ○ Lichte schokken/ bewegingen in delen van het lichaam (2)
- ○ Geen (3)

Page Break

**Indien u slechts twee typen aanvallen heeft, gaat u vriendelijk verder naar de volgende sectie.**

- ○ Aanvalstype C (1)
- ○ Volgende Sectie (2)

Page Break

**Aanvalstype C**

Page Break

Heeft u bij deze aanval schokken over uw hele lichaam?

- ○ Ja (1)
- ○ Nee (2)

Page Break

Hoe vaak valt u als gevolg van dit aanvalstype?

- ○ Bijna altijd/ Altijd (1)
- ○ Vaak (2)
- ○ Soms (3)
- ○ Nooit (4)

Page Break

**Ontstaat er als gevolg van dit aanvalstype een van de volgende letsels?** Kruis  één hokje aan: het meest ernstige.

- ○ Verbranding, diepe snijwonden, breuken  (1)
- ○ Tongbeet of ernstige hoofdpijn  (2)
- ○ Milde verwonding of milde hoofdpijn  (3)
- ○ Geen verwonding  (4)

Page Break

Hoe vaak is er bij dit aanvalstype sprake van urineverlies?

- ○ Bijna altijd/ Altijd (1)
- ○ Vaak (2)
- ○ Soms (3)
- ○ Nooit (4)

Page Break

Als dit aanvalstype gepaard gaat met een bewustzijnsdaling, voelt u dit lang genoeg van te voren aankomen om uzelf te beschermen?

- ○ Bijna altijd/ Altijd (1)
- ○ Soms (2)
- ○ Nooit (3)
- ○ Geen van deze opties: er is geen bewustzijnsverlies bij mijn aanvallen (4)
- ○ Geen van deze opties: mijn aanvallen komen alleen in slaap voor (5)

Page Break

Hoe lang duurt het voor u weer helemaal de oude bent?

- ○ Minder dan 1 minuut (1)
- ○ Tussen 1 en 10 minuten (2)
- ○ Tussen 10 min en 1 uur (3)
- ○ Tussen 1 en 3 uur (4)
- ○ Meer dan 3 uur (5)

Page Break

**Komen bij dit type aanval de volgende verschijnselen voor?** Kruis één hokje aan: het meest ernstige

- ○ Schreeuwen; dwalen en/of ontkleden (1)
- ○ Lichte schokken/ bewegingen in delen van het lichaam (2)
- ○ Geen (3)

**End of Block: Block 1-2 Optionele achtergrondvragen**

---

**Start of Block: Block 6-qoile31p**

**AANWIJZINGEN** Deze vragenlijst gaat over uw gezondheid en dagelijkse bezigheden. **Beantwoord elke vraag** door het juiste cijfer (1, 2, 3…) te omcirkelen. Als u niet zeker weet hoe u moet antwoorden, geef dan a.u.b. het best mogelijke antwoord en schrijf er. Vraag gerust om hulp als u moeite heeft met het lezen of invullen van dit formulier.

Page Break

X→

**Hoe zou u, over het geheel genomen, de kwaliteit van uw leven beoordelen?** Verplaats de schaal naar links voor **( Slechtst Mogelijke Kwaliteit van Leven )** en naar rechts voor **(Best Mogelijke Kwaliteit van Leven).**

- ○ 0 (1)
- ○ 1 (2)
- ○ 2 (3)
- ○ 3 (4)
- ○ 4 (5)
- ○ 5 (6)
- ○ 6 (7)
- ○ 7 (8)
- ○ 8 (9)
- ○ 9 (10)
- ○ 10 (11)

Page Break

**Deel A.** Deze vragen gaan over hoe u zich in de afgelopen 4 weken GEVOELD heeft. Wilt u a.u.b bij elke vraag het antwoord geven dat het best benadert hoe u zich voelde.

Hoe vaak gedurende de afgelopen 4 weken…

| | Altijd 1 (6) | Meestal 2 (5) | Vaak 3 (4) | Soms 4 (3) | Zelden 5 (2) | Nooit 6 (1) |
|---|---|---|---|---|---|---|
| Voelde u zich levenslustig? (1) | ○ | ○ | ○ | ○ | ○ | ○ |
| Had u veel energie? (2) | ○ | ○ | ○ | ○ | ○ | ○ |
| Voelde u zich uitgeput? (3) | ○ | ○ | ○ | ○ | ○ | ○ |
| Voelde u zich moe? (4) | ○ | ○ | ○ | ○ | ○ | ○ |

**Deel A.** Bij het nagaan van de vragen in **Deel A** overweeg de algemene invloed van deze punten op uw leven **in de afgelopen 4 weken.**

In welke mate **maken** de bovenstaande problemen en zorgen in verband met uw energie u in het algemeen **benauwd?**

| | Helemaal niet 1 (1) | Een beetje 2 (2) | Middelmatig 3 (3) | Veel 4 (4) | Zeer Veel 5 (5) |
|---|---|---|---|---|---|
| 1 (1) | ○ | ○ | ○ | ○ | ○ |

Page Break

**Deel B.** Deze vragen gaan over hoe u zich in de afgelopen 4 weken GEVOELD heeft. Wilt u a.u.b bij elke vraag het antwoord geven dat het best benadert hoe u zich voelde.

Hoe vaak gedurende de afgelopen 4 weken…

| | Altijd 1 (6) | Meestal 2 (5) | Vaak 3 (4) | Soms 4 (3) | Zelden 5 (2) | nooite 6 (1) |
|---|---|---|---|---|---|---|
| Was u erg zenuwachtig? (1) | ○ | ○ | ○ | ○ | ○ | ○ |
| Zat u zo in de put dat niets u kon opvrolijken? (2) | ○ | ○ | ○ | ○ | ○ | ○ |
| Voelde u zich rustig en tevreden? (3) | ○ | ○ | ○ | ○ | ○ | ○ |
| Voelde u zich somber en neerslachtig? (4) | ○ | ○ | ○ | ○ | ○ | ○ |
| Was u een gelukkig mens? (5) | ○ | ○ | ○ | ○ | ○ | ○ |

**Deel B.** Bij het nagaan van de vragen in **Deel B** overweeg de algemene invloed van deze punten op uw leven **in de afgelopen 4 weken.**

In welke mate **maken** de bovenstaande problemen en zorgen in verband met uw **emoties** u in het algemeen **benauwd?**

| | Helemaal niet 1 (1) | Een beetje 2 (2) | Middelmatig 3 (3) | Veel 4 (4) | Zeer Veel 5 (5) |
|---|---|---|---|---|---|
| 1 (1) | ○ | ○ | ○ | ○ | ○ |

Page Break

**Deel C.** De volgende vragen gaan over hoe u zich VOELT en over problemen die u met dagelijkse ACTIVITEITEN in de afgelopen 4 weken gehad heef. Wilt u a.u.b bij elke vraag het antwoord geven dat het best benadert hoe u zich voelde

De volgende vraag gaat over hoe u zich VOELT en hoe het met u gaat. **Hoe vaak gedurende de afgelopen 4 weken …**

| | Altijd 1 (6) | Meestal 2 (5) | Vaak 3 (4) | Soms 4 (3) | Zelden 5 (2) | Nooit 6 (1) |
|---|---|---|---|---|---|---|
| Heeft uw gezondheid u beperkt bij uw sociale activiteiten (zoals vrienden of familie bezoeken)? (1) | ○ | ○ | ○ | ○ | ○ | ○ |

De volgende vragen gaan over problemen die u misschien heeft bij bepaalde dagelijkse ACTIVITEITEN. **Hoe lang gedurende de afgelopen 4 weken, heeft uw epilepsie of anti-epileptische medicijnen u problemen gegeven bij…**

| | Heel veel 1 (1) | Veel 2 (2) | Enigszins 3 (3) | Maar weinig 4 (4) | Helemaal niet 5 (5) |
|---|---|---|---|---|---|
| Activiteiten in uw vrije tijd (zoals hobbies, uitgaan)? (1) | ○ | ○ | ○ | ○ | ○ |
| Autorijden (of gebruik van vervoer)? (2) | ○ | ○ | ○ | ○ | ○ |

De volgende vragen gaan over problemen die u misschien heeft bij bepaalde dagelijkse ACTIVITEITEN. **Hoe lang gedurende de afgelopen 4 weken, heeft uw epilepsie of anti-epileptische medicijnen u problemen gegeven bij…**

| | Helemaal geen last van | | | Heel veel last van | | |
|---|---|---|---|---|---|---|
| | 1 | 2 | 3 | 3 | 4 | 5 |
| In welke mate zijn uw werkbeperkingen vervelend voor u? (1) | | | | | | |
| In welke mate zijn uw beperkingen in uw sociale contacten vervelend voor u? (2) | | | | | | |

**Deel C.** Bij het nagaan van de vragen in **Deel C** overweeg de algemene invloed van deze punten op uw leven **in de afgelopen 4 weken.**

In welke mate **maken** de bovenstaande problemen en zorgen in verband met uw d**agelijkse activiteite**n u in het algemeen **benauwd?**

| | Helemaal niet 1 (1) | Een beetje 2 (2) | Middelmatig 3 (3) | Veel 4 (4) | Zeer Veel 5 (5) |
|---|---|---|---|---|---|
| 1 (1) | ○ | ○ | ○ | ○ | ○ |

Page Break

**Deel D.** Deze vragen gaan over problemen met nadenken, lezen, concentratie en geheugen die u mogelijk gehad heeft in de afgelopen 4 weken. Wilt u a.u.b bij elke vraag het antwoord geven dat het best benadert hoe u zich voelde.

**Hoe vaak gedurende de afgelopen 4 weken…**

| | Altijd 1 (6) | Meestal 2 (5) | Vaak 3 (4) | Soms 4 (3) | Zelden 5 (2) | Nooit 6 (1) |
|---|---|---|---|---|---|---|
| Had u moeite met redeneren en het oplossen van problemen (bijvoorbeeld plannen maken, besluiten nemen, nieuwe dingen leren)? (1) | ○ | ○ | ○ | ○ | ○ | ○ |

Heeft u in de afgelopen 4 weken problemen gehad met uw geheugen?

| | Ja, veel (1) | Ja, enigszins (2) | Maar weinig (3) | Nee, Helemaal niet (4) |
|---|---|---|---|---|
| 1 (1) | ○ | ○ | ○ | ○ |

**Hoe vaak gedurende de afgelopen 4 weken, had u....**

| | Altid 1 (6) | Meestal 2 (5) | Vaak 3 (4) | Soms 4 (3) | Zelden 5 (2) | Nooit 6 (1) |
|---|---|---|---|---|---|---|
| In welke mate zijn uw problemen met uw geheugen vervelend (1) | ○ | ○ | ○ | ○ | ○ | ○ |
| Moeite met het concentreren bij het lezen? (2) | ○ | ○ | ○ | ○ | ○ | ○ |
| Moeite om u op één ding tegelijk te concentreren? (3) | ○ | ○ | ○ | ○ | ○ | ○ |

In welke mate zijn uw beperkingen in uw sociale contacten vervelend voor u?

Helemaal geen last van    Heel veel last van

| | 1 | 2 | 3 | 4 | 5 |
|---|---|---|---|---|---|
| 1 (1) | | | | | |

**Deel D.** Bij het nagaan van de vragen in **Deel D** overweeg de algemene invloed van deze punten op uw leven **in de afgelopen 4 weken.**

In welke mate **maken** de bovenstaande problemen en zorgen in verband met uw geestelijk functioneren u in het algemeen **benauwd?**

| | Helemaal niet 1 (1) | Een beetje 2 (2) | Middelmatig 3 (3) | Veel 4 (4) | Zeer Veel 5 (5) |
|---|---|---|---|---|---|
| 1 (1) | ○ | ○ | ○ | ○ | ○ |

Page Break

**Deel E.** Deze vragen gaan over mogelijke problemen in verband met uw epilepsie of uw anti-epilepsie medicatie

Gedurende de afgelopen 4 weken…

| | Helemaal geen last van 1 | 2 | 3 | 4 | Heel veel last van 5 |
|---|---|---|---|---|---|
| In welke mate zijn de lichamelijke bijwerkingen van anti epileptische medicijnen vervelend voor u? (1) | | | | | |
| In welke mate zijn de psychische bijwerken van anti epileptische medicijnen vervelend voor u? (2) | | | | | |

Hoe bezorgd bent u dat de medicijnen die u inneemt slecht voor u kunnen zijn als u ze langdurig inneemt?

| | Heel bezorgd (1) | enigszins bezorgd (2) | niet zo erg bezorgd (3) | Helemaal niet bezorgd (4) |
|---|---|---|---|---|
| 1 (1) | ○ | ○ | ○ | ○ |

**Deel E.** Bij het nagaan van de vragen in **Deel E** overweeg de algemene invloed van deze punten op uw leven **in de afgelopen 4 weken.**

In welke mate **maken** de bovenstaande problemen en zorgen  in verband met de gevolgen van uw medicatie u in het  algemeen **benauwd?**

| | Helemaal niet 1 (1) | Een beetje 2 (2) | Middelmatig 3 (3) | Veel 4 (4) | Zeer Veel 5 (5) |
|---|---|---|---|---|---|
| 1 (1) | ○ | ○ | ○ | ○ | ○ |

Page Break

**Deel F.** Deze vragen gaan over hoe u zich VOELT over uw aanvallen in de afgelopen 4 weken. Wilt u a.u.b bij elke vraag het antwoord geven dat het best benadert hoe u zich voelde.

**Hoe vaak gedurende de afgelopen 4 weken…**

| | Altijd 1 (6) | Meestal 2 (5) | Vaak 3 (4) | Soms 4 (3) | Zelden 5 (2) | Nooit 6 (1) |
|---|---|---|---|---|---|---|
| Maakte u zich er zorgen over weer een epileptische aanval te krijgen? (1) | ○ | ○ | ○ | ○ | ○ | ○ |

Hoe bang bent u ervoor in de komende 4 weken een epileptische aanval te hebben?

| | Heel bang (1) | Enigszins bang (2) | Niet erg bang (3) | Helemaal niet bang (4) |
|---|---|---|---|---|
| 1 (1) | ○ | ○ | ○ | ○ |

Maakt u zich zorgen dat u zichzelf bezeert tijdens een epileptische aanval?

| | Veel zorgen (1) | Een beetje zorgen (2) | Helemaal geen zorgen (3) |
|---|---|---|---|
| 1 (1) | ○ | ○ | ○ |

Hoe bezorgd bent u dat u in de komende 4 weken in verlegenheid gebracht zal worden of dat u andere problemen in uw sociale contacten zal hebben tengevolge van een epileptische aanval?

| | Heel bezorgd (1) | Enigszins bezorgd (2) | Niet zo erg bezord (3) | Helemaal niet bezorgd (4) |
|---|---|---|---|---|
| 1 (1) | ○ | ○ | ○ | ○ |

In welke mate zijn uw epileptische aanvallen vervelend voor u?

| | Helemaal geen last van | | | Heel veel last van | |
|---|---|---|---|---|---|
| | 1 | 2 | 3 | 4 | 5 |
| 1 (1) | | | | | |

**Deel F.** Bij het nagaan van de vragen in **Deel F** overweeg de algemene invloed van deze punten op uw leven **in de afgelopen 4 weken.**

In welke mate **maken** de bovenstaande problemen en zorgen in verband met uw **aanvallen** u in het algemeen **benauwd?**

| | Helemaal niet 1 (1) | Een beetje 2 (2) | Middelmatig 3 (3) | Veel 4 (4) | Zeer Veel 5 (5) |
|---|---|---|---|---|---|
| 1 (1) | ○ | ○ | ○ | ○ | ○ |

**Deel G.** De volgende vraag gaat over hoe u zich VOELT met betrekking tot uw algemene levenskwaliteit. Voor elke vraag, gelieve het antwoord aan te duiden dat het dichtst komt bij hoe u zich voelde.

Hoe was de **KWALITEIT VAN UW LEVEN** gedurende de afgelopen 4 weken (dat wil zeggen: hoe ging het met u)?

| | Heel goed: kon nauwelijks beter | | Vrij goed | | Ongeveer even goed als slecht | | Vrij slecht | | Heel slecht: kon nauwelijks slechter |
|---|---|---|---|---|---|---|---|---|---|
| | 0 | 1 | 2 | 3 | 3 | 4 | 5 | | |
| **Verplaats de schaal om een nummer te kiezen** (2) | | | | | | | | | |

Page Break

**Deel G.** Bij het nagaan van de vragen **1 en 36 in deel G** (op bladzijde 1 en op deze bladzijde), overweeg de algemene invloed van uw levenskwaliteit gedurende **de afgelopen 4 weken.**

In welke mate **maakt** de stand van uw **levenskwaliteit** u in het algemeen **benauwd?**

| | Helemaal niet 1 (1) | Een beetje 2 (2) | Middelmatig 3 (3) | Veel 4 (4) | Zeer Veel 5 (5) |
|---|---|---|---|---|---|
| 1 (1) | ○ | ○ | ○ | ○ | ○ |

Page Break

**Deel H.** Hoe goed of slecht denkt u dat uw gezondheid is? Op de thermometer-schaal hieronder staat 100 voor de best denkbare gezondheidstoestand en 0 voor de slechtst denkbare gezondheidstoestand. Geef a.u.b. aan wat u van uw gezondheid vindt door een cijfer op de schaal te omcirkelen. **Beschouw bij het beantwoorden van deze vraag uw epilepsie als onderdeel van uw algehele gezondheid.** Schrijf uw antwoord in het tekstvak.

________________________________________________________________

________________________________________________________________

________________________________________________________________

________________________________________________________________

________________________________________________________________

---

**Deel I.** Rekening houdend met **ALLE** vragen die u heeft beantwoord, geef de met uw epilepsie samenhangende gebieden aan, die **NU** voor uw belangrijk zijn.
**Geef de volgende onderwerpen een nummer 1 tot 7, waarbij 1 het belangrijkste en 7 het minst belangrijke onderwerp weergeeft. Gebruik elk cijfer slechts eenmaal.**
______ A. Energie (vermoeidheid) (1)
______ B. Emoties (stemming) (2)
______ C. Dagelijkse activiteiten (werk, autorijden, sociaal) (3)
______ D. Geestelijke activiteit (denken, concentratie, geheugen) (4)
______ E. Gevolgen van de medicatie (fysiek, geestelijk) (5)
______ F. Angst voor aanvallen (gevolgen van aanvallen) (6)
______ G. Algemene levenskwaliteit (7)

End of Block: Block 6-qoile31p

Start of Block: Block 7 - ICECAP A OVER UW ALGEHELE KWALITEIT VAN LEVEN

**De volgende vragenlijst gaat over uw algehele kwaliteit van leven.** Geef hieronder aan welke uitspraken uw algehele kwaliteit van leven op dit moment het beste beschrijven. Plaats hiervoor een vinkje in **ÉÉN** vak in elk van de vijf groepen hieronder.

---

Page Break

**1. Op mijn plek en veilig voelen**

○ Op **alle** gebieden van mijn leven ben ik in staat me op mijn plek en veilig te voelen (1)

○ Op **veel** gebieden van mijn leven ben ik in staat me op mijn plek en veilig te voelen (2)

○ Op **enkele** gebieden van mijn leven ben ik in staat me op mijn plek en veilig te voele (3)

○ Op **geen enkel** gebied van mijn leven ben ik in staat me op mijn plek en veilig te voelen (4)

---

Page Break

**2. Liefde, vriendschap en ondersteuning**

- ◯ Ik ben in staat om **veel** liefde, vriendschap en ondersteuning te hebben (1)
- ◯ Ik ben in staat om **best veel** liefde, vriendschap en ondersteuning te hebben (2)
- ◯ Ik ben in staat om **een beetje** liefde, vriendschap en ondersteuning te hebben (3)
- ◯ Ik ben **niet** in staat om liefde, vriendschap en ondersteuning te hebben (4)

Page Break

**3. Onafhankelijk zijn**

- ○ Ik ben in staat om **volledig** onafhankelijk te zijn (1)
- ○ Ik ben in staat om onafhankelijk te zijn in **veel** dingen (2)
- ○ Ik ben in staat om onafhankelijk te zijn in **enkele** dingen (3)
- ○ Ik ben in het geheel **niet** in staat onafhankelijk te zijn (4)

Page Break

**4. Prestaties en vooruitgang**

○ Ik ben in **alle** aspecten van mijn leven in staat om te presteren en vooruitgang te boeken (1)

○ Ik ben in **veel** aspecten van mijn leven in staat om te presteren en vooruitgang te boeken (2)

○ Ik ben in **enkele** aspecten van mijn leven in staat om te presteren en vooruitgang te boeken (3)

○ Ik ben in **geen enkel** aspect van mijn leven in staat om te presteren en vooruitgang te boeken (4)

Page Break

**5. Plezier maken en genieten**

- ○ Ik ben in staat om **veel** plezier te maken en te genieten (1)
- ○ Ik ben in staat om **best veel** plezier te maken en te genieten (2)
- ○ Ik ben in staat om **een beetje** plezier te maken en te genieten (3)
- ○ Ik ben **niet** in staat om plezier te maken en te genieten (4)

We werken ook aan een ander onderzoek, waarvan het hoofddoel is om een impactmaat voor de epilepsiezorg te ontwikkelen en deze maat in Nederland te implementeren om de huidige zorg te monitoren en te verbeteren. Als u wilt deelnemen aan dit onderzoek, typ dan "ja". Hier is de beschrijving van dit onderzoek: Dit onderzoek heeft als hoofddoel het ontwikkelen van een impactmaat voor de epilepsiezorg en het implementeren van deze maat in Nederland om de bestaande zorg te monitoren en verbeteren.

________________________________________________________________

Page Break

End of Block: Block 7 - ICECAP A OVER UW ALGEHELE KWALITEIT VAN LEVEN
