# Supplementary material for: The Economic and societal burden associated with drug-resistant epilepsy in the Netherlands: an AIM@EPILEPSY burden-of-disease study protocol
Source: BMJ Open. 2025 Jul 25;15(7):e095123. doi: 10.1136/bmjopen-2024-095123 (PMC12306250; doi:10.1136/bmjopen-2024-095123)
Supplement: Supplementary file 2 [file bmjopen-2024-095123.reviewer_comments.pdf]

PEER REVIEW HISTORY

BMJ Open publishes all reviews undertaken for accepted manuscripts. Reviewers are asked to complete a checklist review form and are provided with free text boxes to elaborate on their assessment. These free text comments are reproduced below.

ARTICLE DETAILS

Title (Provisional)

The economic and societal burden associated with drug-resistant epilepsy in the Netherlands: An AIM@EPILEPSY burden-of-disease study protocol.

Authors

Elabbasy, Darin; Evers, Silvia; Majoie, Marian H.J.M.; Schijns, Olaf Elisabeth Maria Ghislaine; M'Rabet, Laura; van Kranen-Mastenbroek, VHJM; Eekers, Daniëlle B P; Houben, Ruud; Hendriks, Marc; Colon, Albert; van Mastrigt, Ghislaine APG

VERSION 1 - REVIEW

**Reviewer**	**1**
**Name**	**Khoo, Ching Soong**
**Affiliation**	**Universiti Kebangsaan Malaysia**
**Date**	**31-Jan-2025**
**COI**	**None**

I applaud the authors for their laborious work and effort.

I do have the following comments or suggestions:

1. In the Introduction Section, line 93, I suggest that the authors briefly talk about neuromodulation (such as VNS, DBS, etc.) as a treatment option for patients with DRE.

2. Recruitment criteria, line 176: Please define who caregivers are.

3. Baseline epilepsy data, line 242: Please include duration of epilepsy, and types of ASMs (monotherapy v.s. polytherapy), and activities of daily living (dependent v.s. independent). For caregivers, please include their relationship to PWE (patients living with epilepsy), hours spent with caregiving, and any underlying medical conditions of the caregivers. (Please refer to this paper DOI: 10.1016/j.yebeh.2024.110244)

**Reviewer**	**2**
**Name**	**Fong , Si Lei**
**Affiliation**	**Universiti Malaya**

**Date**	**26-Feb-2025**
**COI**	**None**

This is a proposal and not a completed study with results.

**Reviewer**	**3**
**Name**	**Tabrizi, Nasim**
**Affiliation**	**MazUMS**
**Date**	**03-May-2025**
**COI**	**None**

Given the disparity in the burden and impact on quality of life among patients with acute symptomatic seizures, first unprovoked seizures, and epilepsy, it is recommended that only patients with epilepsy be enrolled in the study.

The patient's statement of a diagnosis of epilepsy appears to be inadequate for confirming the diagnosis. It is strongly recommended that patients with a confirmed diagnosis of epilepsy (one of the three conditions delineated by the International League Against Epilepsy [ILAE]) seek approval from a neurologist to participate in the study.

VERSION 1 - AUTHOR RESPONSE

Reviewer 1:

Dr. Ching Soong Khoo, Universiti Kebangsaan Malaysia

Comments to the Author: I applaud the authors for their laborious work and effort.

Thank you, Dr. Ching for your kind comment.

I do have the following comments or suggestions:

1. **Comment**: in the Introduction Section, line 93, I suggest that the authors briefly talk about neuromodulation (such as VNS, DBS, etc.) as a treatment option for patients with DRE.

Respnse: Thank you Dr. Ching Soong Khoo for your comment. We have now briefly mentioned neuromodulation in its role in the management of DRE.

Please see line 101- 106

> *"Another possible intervention for patients ineligible for curative (re)surgery is neuromodulation, a palliative intervention aimed usually to prevent or reduce ictal events. These includes invasive methods such as vagus nerve stimulation (VNS), deep*

brain stimulation(DBS) and responsive neurostimulation, as well as noninvasive techniques including transcutaneous vagus nerve stimulation(tVNS), transcranial magnetic stimulation (TMS), transcranial direct current stimulation (tDCS) and trigeminal nerve stimulation (TNS)"

2. **Comment** : recruitment criteria, line 176: Please define who the caregivers are.

Response: Thank you for your comment. With caregivers, we refer to informal caregivers providing unpaid assistance, such as help with personal hygiene, meal preparation or mobility, to a person with epilepsy who exists within their immediate social circle. It typically involves unpaid support from family members, friends or neighbors to meet daily living needs and provide emotional comfort. We have now adjusted this in the text of the protocol and added clarification to the survey questionnaire.

Please see lines 176-180

> *"With caregivers we refer to unpaid informal carers providing unpaid assistance, such as help with personal hygiene, meal preparation or mobility, to a person with epilepsy who exists within their immediate social circle…"*

3. **Comment**: Baseline epilepsy data, line 242: Please include duration of epilepsy, and types of ASMs (monotherapy v.s. polytherapy), and activities of daily living (dependent v.s. independent). For caregivers, please include their relationship to PWE (patients living with epilepsy), hours spent with caregiving, and any underlying medical conditions of the caregivers. (Please refer to this paper DOI: 10.1016/j.yebeh.2024.110244)

Response: Thank you for your comment. We have updated the baseline questions to include the year of initial diagnosis, current seizure status (active vs. seizure-free), and independence in activities of daily living.

See lines 268-270

> *"Additionally, information regarding the year of initial diagnosis, current seizure status (active vs. seizure-free), and level of independence in performing activities of daily living. will be collected"*

For the caregiver survey, we added questions to clarify the caregiver's relationship to the care recipient, the average hours spent per week providing care, and whether the caregiver has any underlying medical conditions and referred to the suggested study.

Please see lines 271- 275

"*For caregivers, the baseline questionnaire will collect data on age, gender, and employment status. In addition to questions related to epilepsy care recipients, and in line with other studies investigating the burden of care for epilepsy, we will collect information such as the caregiver's relationship to the care recipient, the average hours spent per week providing care, and whether the caregiver has any underlying medical conditions*[6 29] *(See appendix C: Questionnaires)"*

Reviewer: 2

Dr. Si Lei Fong , Universiti Malaya

Comments to the Author:

This is a proposal and not a completed study with results.

Response: Thank you, Dr. Fong, for your comment. While it is correct that this submission is a research protocol rather than a completed study with results, my co-author and I decided on publishing the protocols is as it ensures transparency, facilitates peer review of study methodology, and contributes to the reproducibility and credibility of future research outcomes.

Reviewer 3:

Dr. Nasim Tabrizi, MazUMS

Comments to the Author: Given the disparity in the burden and impact on quality of life among patients with acute symptomatic seizures, first unprovoked seizures, and epilepsy, it is recommended that only patients with epilepsy be enrolled in the study.

Response: Thank you Dr Nasim for your feedback. We agree with the recommendation, and to clarify, our study includes only individuals with a diagnosis of epilepsy.

Within this population, one of our key objectives is to better understand the burden on individuals with drug resistant epilepsy, whose data remains fragmented, particularly in the Netherlands. To this end, our survey includes specific questions regarding seizure frequency, current seizure status, most recent seizure experience, and treatment history, allowing us to capture the severity and complexity of their condition.

Please see lines 167- 185

> "This study will invite participants with the following criteria:
>
> 1) Persons with epilepsy:
>
> • Aged 18 and above, primarily residing in the Netherlands, fluent in Dutch and can consent to participate.
>
> • A confirmed diagnosis of epilepsy by a neurologist (self-reported by study participants)…"

Comment: The patient's statement of a diagnosis of epilepsy appears to be inadequate for confirming the diagnosis. It is strongly recommended that patients with a confirmed diagnosis of epilepsy (one of the three conditions delineated by the International League Against Epilepsy [ILAE]) seek approval from a neurologist to participate in the study.

Thank you for raising this point. To address this, our survey includes specific questions asking participants to indicate when they were diagnosed with epilepsy and how often they are seen by a neurologist. While we recognize the limitations of self-reported data, these items are intended to ensure to an extent that participants have a confirmed diagnosis aligned with the International League Against Epilepsy (ILAE) criteria. We think that requiring formal neurologist approval for participation would significantly reduce feasibility and accessibility. However, we have clearly stated in the survey that eligibility requires a diagnosis confirmed by a treating medical specialist in the Netherlands.
